# A Collection and Analysis of Simplified Data for a Better Understanding of the Complex Process of Biofilm Inactivation by Ultraviolet and Visible Irradiation

**DOI:** 10.3390/microorganisms13092048

**Published:** 2025-09-03

**Authors:** Martin Hessling, Wendy Meulebroeck, Beatrix Alsanius

**Affiliations:** 1 Institute of Medical Engineering and Mechatronics, Technische Hochschule Ulm (University of Applied Sciences), Albert-Einstein-Allee 55, 89081 Ulm, Germany; 2 Brussels Photonics (B-PHOT), Department of Applied Physics and Photonics, Vrije Universiteit Brussel, Pleinlaan 2, 1050 Brussels, Belgium; wendy.meulebroeck@vub.be; 3 Department of Biosystems and Technology, Campus Alnarp, Swedish University of Agricultural Sciences, P.O. Box 190, SE-23422 Lomma, Sweden; beatrix.alsanius@slu.se

**Keywords:** biofilm, prevention, reduction, ultraviolet radiation, visible light

## Abstract

Biofilms are communities of microorganisms that pose a problem in many areas, including the food industry, drinking water treatment, and medicine, because they can contain pathogens and are difficult to eliminate. For this reason, the possibility of biofilm reduction by ultraviolet (UV) or visible light was investigated using data from published reports. Results for different applications, spectral ranges, and microorganisms were compared by performing MANOVA tests. Approximately 140 publications were found that dealt with the irradiation of water or surfaces for biofilm reduction or reduction in biofilm formation. Irradiation of surfaces with UV or visible light in the spectral range 200–525 nm had a positive effect on biofilm reduction and reduction in biofilm formation, although the results for irradiation of water were conflicting. Most investigations were carried out on *P. aeruginosa* biofilms, but other Gram-positive and Gram-negative bacteria, as well as some fungi and their biofilm sensitivities to irradiation, were also analyzed. Limited data were available for the UVB (280–315 nm) and UVA (315–400 nm) range. Most experiments to date have been carried out in the UVC (100–280 nm) or in the visible violet/blue spectral (400–500 nm) range, with the UVC range being 2–3 orders of magnitude more efficient in terms of applied irradiation dose. Other quantitative statements were difficult to make as the results from the different working groups were highly scattered. Irradiation can reduce the microorganisms in biofilms but does not completely remove biofilms. New biofilm formation can at least be delayed by surface irradiation. Whether it is also possible to prevent the formation of new biofilms in the long term is open to question. Which irradiation wavelengths are optimal for anti-biofilm measures is also still unclear.

## 1. Introduction

Biofilms are aggregates of microorganisms in which cells are embedded in a self-produced matrix of extracellular polymeric substances that adhere to surfaces [1]. These microorganisms can include algae, archaea, bacteria, fungi, and protozoa, and they can form in a wide range of environments, from natural ecosystems to industrial settings, medical devices, and agricultural systems [2]. Biofilms can develop on plant surfaces, in livestock water troughs, in food processing facilities, and in irrigation systems [3]. These biofilms have a significant impact on human and animal health, pose food safety challenges, and contaminate drinking and irrigation water supplies, but they can also be beneficial for plant growth or water treatment processes [4,5]. Based on a market analysis using values from 2019, it was estimated that biofilms have a global economic significance of more than $5000 billion a year, with further market sector breakdowns of $324 billion (food and agriculture) and $117 billion (water and wastewater) [4].

The biofilm matrix is a dynamic space with continuous production and degradation of all extracellular polymeric substances (EPS). The many structural components are produced, transformed, and degraded by various extracellular matrix housekeeping enzymes, by the biofilm community. The evolution of the matrix takes place in response to the amount and nature of nutrients available and the environmental conditions they encounter, such as hydrodynamic shear stress, pressure, salt content, temperature, and light regime [6].

This review summarizes the potential biofilm-reducing effect of UV radiation and short-wave visible light. The ultraviolet spectral range is divided into UVC (100–280 nm), UVB (280–315 nm), and UVA (315–400 nm), while visible light covers the spectral range from 380 to 780 nm. Both UV radiation and short-wave violet and blue light (<500 nm) are known to be capable of inactivating microorganisms. The most important mechanisms of action are the destruction of DNA, in particular by UVC radiation, or the generation of intracellular reactive oxygen species (ROS), which can attack all cell structures from within with subsequent cell death. The latter is the main basis for the antimicrobial effect of visible light, but also of UVA radiation [7,8,9,10].

This known effect of light or radiation on planktonic microorganisms is the basis for the approach analyzed here of using UV radiation and visible light against biofilms, as these are formed by microorganisms and contain microorganisms.

Several state-of-the-art studies, including a recent review by Gora et al. [11], already provided a preliminary understanding of the impact of irradiation in the UV (200–400 nm) and VIS (380–780 nm) spectral regions on biofilms and biofilm formation. However, there are many open questions, such as the following:

- Does the irradiation of water reduce or prevent biofilm formation in water works and water distribution systems?
- Is it possible to prevent biofilm formation by irradiation in the long term (weeks or months) and what are the best parameters?
- Is biofilm prevention or reduction possible by all UV and VIS wavelengths?
- Which irradiation wavelength is the best?
- Are multi-species biofilms more irradiation resistant than single species biofilms?
- Are cells in biofilms more radiation resistant than planktonic cells?
- Which mathematical model describes the relation between irradiation and biofilm-reduction best? Is there a maximum reduction that cannot be increased even by higher irradiation doses?
- Is there an influence of the substrate below the biofilm?
- Are there differences in the biofilm sensitivity towards irradiation between microorganisms like bacteria or fungi or even between Gram+ and Gram- bacteria?

To answer these questions, an extensive literature research will be performed that hopefully goes beyond the excellent collection by Gora et al. [11]. Data extracted from the retrieved reports are to be analyzed to answer some of these questions.

## 2. Data Collection and Analysis

### 2.1. Data Collection

Literature research with Google Scholar and Pubmed with different combinations of terms like “biofilm”, “irradiation”, “illumination”, “inactivation”, “reduction”, “ultraviolet”, “UVC”, “UVB”, “UVA”, “visible light”, “violet”, “blue” was performed. References in the retrieved literature were also checked for their suitability to be included in this review.

Excluded were results that were gained by combination of irradiation with other disinfection measures, e.g., heat, chemical disinfectants like H_2_O_2_, ozone, chlorine, additional photosensitizer or TiO_2_. Also ignored were bacterial monolayers and bacterial colonies on agar, when irradiated with visible light or UVA radiation, as agar might contain photosensitizer like riboflavin (e.g., in lysogeny broth or yeast extract peptone dextrose agar) and therefore might influence the photoinactivation [12].

Recorded were information on physical biofilm properties before and after irradiation, including cell numbers inside the biofilm or biofilm thickness, but no biochemical data or changes in biofilm composition or color or microbial gene expression. If results at different temperatures were available, the data obtained nearest to room temperature was taken.

If quantitative data on biofilm irradiation was available, up to four data points were taken from a single study for each wavelength and microorganism and sometimes substrate material, like irradiation doses for four different cell log-reductions. In the case that the authors did not give values in the paper or supplementary material, the data was obtained from (enlarged) figures, if available.

### 2.2. Data Analysis

Collected and analyzed were involved microorganisms, biofilm reduction—usually as biofilm cell reduction—irradiation parameters like wavelength, intensity and dose, biofilm age and biofilm substrate in the first step. These data were sorted into different tables according to diverse applications. Results from different authors but for the same species in different spectral regions were also graphically combined if there were at least five data points of at least three different publications. An exponential function—or a straight line in a semi-logarithmic representation—was fitted to all obtained data as first approximation and typical inactivation behavior [13]. In addition, the often applied inactivation models “Weibull” [14] and “log-lin + tail” [15], which both exhibit reduced inactivation for longer irradiation procedures, were also fitted to the data. The intention was to determine which model best described the collected data. This was assessed by RMSE (residual mean squared errors) as calculated by the software Bioinactiavtion (version 4) [16,17].

Statistical tests were conducted to assess whether Gram-positive and Gram-negative bacteria or fungi differed from each other in their sensitivity to radiation and whether the material under the biofilm had an influence. Biofilm irradiation data were sorted by spectral range, irradiation dose, biofilm reduction, and microorganism and analyzed for normal distribution as this is often a requirement for subsequent steps. Here Kolmogorov–Smirnov tests were applied, followed by Wilks’ lambda tests (WL), which is a common MANOVA (Multiple analysis of variance) technique, with the advantage that it is quite robust against violations of the normal distribution [18,19,20,21]. The MANOVA tests Pillai’s trace (PT), Hotteling–Lawley trace (HL) and Roy’s maximum root (RM) were also carried out for comparison. All tests were performed with the online software Statistics Kingdom (version of November 2017) [22]. The same statistical tests were performed to determine whether the irradiation sensitivity of mono- and multi-species biofilms differed and whether the substrate on which the biofilm grew had an influence.

## 3. Results

About 140 publications that met the above-mentioned criteria were collected and analyzed. Most data were found on *Pseudomonas aeruginosa* irradiated by UVC or visible (violet or blue) light. Table 1 provides an overview of all about 50 microorganisms and their taxonomic classes for which biofilm irradiation results were retrieved.

The published investigations were divided into three categories and are therefore presented here in three separate tables:

1. Water irradiation for biofilm prevention/delay on different surfaces (Table 2);
2. Surface irradiation for the prevention/delay of new biofilms (Table 3);
3. Biofilm irradiation for the reduction in existing biofilms (Table 4).

### 3.1. Water Irradiation for Biofilm Prevention/Delay

Table 2 presents the experimental details and results for the irradiation of drinking or waste water for biofilm prevention or delay as published in more than 20 studies. Five reports investigated water contaminated with *Pseudomonas aeruginosa*, but most of these studies applied the present natural water microbiome. The typical irradiation sources were 254 nm low-pressure mercury vapor lamps. However, some other UV wavelengths between 220 und 290 nm were also investigated. Studies with longer irradiation wavelengths irradiation in the UVB, UVA, or even visible light spectrum were not published.

Half of these studies reported a positive effect of the UV irradiation, but the other half observed no effect on biofilm prevention or even an increase in biofilm formation. This might be caused by different water qualities and organic compounds but was not investigated here any further.

**Table 2:** Irradiation of water for biofilm prevention or delay. (“red” background: no biofilm-reducing effect of irradiation—“green” background: biofilm-reducing effect of irradiation; PC: polycarbonate, PE: polyethylene, PS: polystyrene, PVC: polyvinylchloride).

Reference	Irradiation Wavelength, Irradiance, Dose	Microorganisms	Biofilm Age, Thickness, Cells	Biofilm Substrate	Reduction
[23]	254 nm, 0.003 mJ/cm^2^	natural microbiome	24 h–38 d, ≈10^7^ cells/cm^2^	steel, cement (in drinking water)	no significant biofilm reduction by water irradiation
[24]	254 nm, 40 mJ/cm^2^	natural microbiome	4 w–6 m, ≈10^6^–10^7^ cells/cm^2^	PVC, PE, steel, copper (in drinking water)	no significant biofilm reduction by water irradiation
[25]	254 nm, 40 mJ/cm^2^	natural microbiome	20 w, ≈100 µg dry weight/cm^2^	membrane (in water)	water irradiation reduced biofilm formation
[26]	254 nm	natural microbiome	up to 30 d, ≈10^5^–10^6^ cells/cm^2^	PVC, steel (in drinking water)	no significant biofilm reduction by water irradiation
[27]	254 nm, up to 80 mJ/cm^2^	natural microbiome	19 d, ≈10^5^ cells/cm^2^	polyamide membrane (in waste water)	water irradiation increased biofilm formation
[28]	254 nm, up to 259 mJ/cm^2^	natural microbiome	2 h, 30 d, ≈10^6^ cells/cm^2^	PVC (in drinking water)	no significant biofilm reduction by water irradiation (nutrient availability in UV-irradiated water higher; no effect on biofilm density in the long run)
[29]	254 nm, up to 150 mJ/cm^2^	*P. aeruginosa*	24 h	PS	higher UVC doses led to stronger biofilm formation
[30]	254 nm	mixture of *P. aeruginosa*, *E. coli*, *Flavobacterium breve*, *Aeromonas hydrophila*	up to 72 h, ≈10^5^ cells/cm^2^	PC	water irradiation reduced biofilm formation (difference ≤ 1 log/cm^2^ after 72 h)
[31]	254 nm, 40 mJ/cm^2^	natural microbiome	4 w–6 m, ≈10^5^–10^6^ cells/cm^2^	PC, iron	no biofilm reduction by UV alone
[32]	254 nm, 40 mJ/cm^2^	natural microbiome	3 m, ≈5 × 10^4^–7 × 10^6^ cells/cm^2^	steel, copper	depending on detection technique and parameters no biofilm reduction
[33]	254 nm, 1900 mJ/cm^2^ every 30 min (pulsed)	natural microbiome	32 d	hollow fiber membrane (surface water)	water irradiation prevented biofilm formation for 32 days
[34]	254 nm, 49 mW/cm^2^; up to 29,000 mJ/cm^2^	natural microbiome	3 h	hollow fiber membrane (in waste water)	water irradiation reduced biofilm formation
[35]	254 nm; broadband UVC (MP Hg)	natural microbiome	up to 200 d	unknown coupons (in drinking water)	water irradiation did not lead to a biofilm decrease; especially for broadband UVC there even seemed to be an increased biofilm formation
[36]	broadband UVC (MP Hg), 80 mJ/cm^2^	natural microbiome	up to 4 m, 120–230 µm; 10^6^–10^7^ cells/cm^2^	membranes (in brackish water)	water irradiation reduced biofilm formation
[37]	220 nm, 260 nm, 280 nm, broadband UVC (MP Hg), up to 8.8 mJ/cm^2^	isolates of natural microbiome	24 h–38 d, ≈10^9^ cells/cm^2^	glass, PP in sea water	280 nm water irradiation decreased biomass; other wavelength had no larger effect or even increased biofilm formation
[38]	220 nm, 239 nm, 254 nm, 260 nm, 270 nm, 280 nm, broadband UVC (MP Hg), up to ≈15 mJ/cm^2^	*P. aeruginosa*	up to 34 h	PS MTP	water irradiation reduced biofilm formation; 254 nm, 270 nm and broadband UVC were most effective (higher bacterial concentration led to stronger biofilm formation)
[39]	broadband UVC (MP Hg), ≈0.135 mW/cm^2^, up to 8 mJ/cm^2^; filtered UV > 295 nm, ≈0.045 mW/cm^2^, up to 40 mJ/cm^2^;	*P. aeruginosa*	up to 9 d	plastic MTP	water irradiation reduced biofilm formation (UV pretreatment of bacteria resulted in lower concentrations and reduced biofilm formation; in the long term: the UV treatment was unable to prevent biofilm formation)
[40]	broadband UVC (MP Hg)	*P. aeruginosa*	24 h, 15–20 µm	glass, PVC, steel (in drinking water)	≥99.9% biofilm volume reduction by water irradiation (decisive for biofilm formation: bacterial concentration, but not whether bacteria were previously irradiated)
[41]	broadband UVC (MP Hg), 137 mJ/cm^2^	natural microbiome	≈10 µm	membrane in brackish water	irradiation (alone) did not lead to biofilm reduction
[42]	254 nm, 42 mJ/cm^2^	natural microbiome	(20 m)	PE in drinking water	water irradiation reduced biofilm formation
[43]	254 nm; broadband UVC (MP Hg), 40 mJ/cm^2^	natural microbiome	up to 170 d	membrane in water	water irradiation reduced biofilm formation (membrane running time was increased by factor 6x)
[44]	275 nm, up to ≈30 mW/cm^2^ (pulsed and continuous)	natural microbiome	up to 11 d	membrane in water	water irradiation reduced biofilm formation
[45]	278 nm, 2 mJ/cm^2^	natural microbiome	up to 15 d, 10^8^–10^9^ cells/cm^2^	membrane (in tap water)	water irradiation reduced biofilm formation
[46]	254 nm; 283 nm, 40 mJ/cm^2^	natural microbiome	5 d	PC in waste water	water irradiation reduced/retarded biofilm formation (no significant difference between irradiated and not irradiated water in the long run)
[47]	280 nm; 40 mJ/cm^2^	*E. coli*	5 d	membrane in contaminated water	water irradiation reduced/delayed biofilm formation—higher UV doses led to more biofilm

### 3.2. Surface Irradiation for Biofilm Prevention

About 30 studies addressed the question of whether the irradiation of surfaces that were biofilm-free at the beginning of the experiment reduced or delayed or even prevented biofilm formation. These are listed in Table 3. Many of these studies focused on the prevention of biofouling on surfaces exposed to seawater. Other authors investigated biofilm prevention in medical applications or in the food or drinking water sector. Here, too, irradiation was mainly carried out in the UVC spectral range at 254 nm, but visible light wavelengths up to 625 nm (red) or even up to 970 nm (infrared) were applied in around 10 studies. The irradiation was partly continuous and partly pulsed.

With only one exception, a positive effect of irradiation on new biofilm formation was observed, i.e., biofilm formation was at least slower under irradiation with wavelengths between 222 and 450 nm. Some authors also reported that biofilm formation no longer occurred above certain irradiation intensities. Values between 0.1 and 100 µW/cm^2^ were given as a sufficient UVC irradiation for the total prevention of biofilm formation [48,49,50,51,52,53,54]. However, some authors still observed biofilm formation at even higher UVC irradiances [55,56,57,58], and on closer inspection, slight biofilm formation could be recognized in some studies that claimed biofilm prevention thresholds for UVC irradiation.

Torkzadeh et al. suggested a mathematical model for (*E. coli*) biofilm formation under different UVC irradiations. The higher the irradiation intensity the slower the biofilm formation, but according to this model it is never zero [58,59]. If this is true, there might be no overall UVC irradiation intensity that totally prevents biofilm formation.

Other spectral regions were less investigated. There was only one UVA study [60] and a few in the visible light region above 400 nm. To avoid often observed confusion, it should be mentioned that the spectral range 380–400 nm is UVA and also visible violet light by definition [61]. However, wavelengths above 400 nm are only visible light and no UV as sometimes erroneously stated by LED manufacturers. Vollmerhausen et al. observed total biofilm prevention for 2.5 mW/cm^2^ UVA and Butement et al. the same for 160 mW/cm^2^ of 405 nm violet light [60,62]. However, the situation might be similar as in the UVC range, with just low—but not zero—biofilm formation at these irradiances.

Whether UVC radiation or visible violet or blue light is better for preventing or delaying biofilm formation cannot be deduced from the widely varying irradiation conditions and results. However, red light and infrared irradiation exhibited no recognizable influence on biofilm formation in any of the presented studies.

**Table 3:** Irradiation of surfaces for biofilm prevention or delay. (“red” background: no biofilm-reducing effect of irradiation—“green” background: biofilm-reducing effect of irradiation; PC: polycarbonate, PE: polyethylene, PS: polystyrene, PVC: polyvinylchloride, MTP: microtiter plate).

Reference	Irradiation Wavelength, Irradiance, Dose	Microorganisms	Biofilm Age, Thickness, Cells	Biofilm Substrate	Reduction
[55]	222 nm, 0.236 mW/cm^2^; up to 354 mJ/cm^2^	*P. aeruginosa*, *S. aureus*	24 h, 48 h, ≈20 µm	steel	biofilm formation observed under continuous far-UVC irradiation, but formation much slower than biofilm formation in the dark
[50]	254 nm, ≤0.0008 mW/cm^2^	natural microbiome	5 w–4 m	copper, silicone, epoxy	continuous irradiation prevented biofilm formation on most materials; 1 min irradiation per day reduced biofilm formation
[63]	254 nm, 1.15 mW/cm^2^; up to 18.4 mJ/cm^2^ per vehicle run	natural microbiome	1 m	steel, copper (in seawater)	successful after two weeks, but biofilm increase after 4 weeks (mobile UVC vehicle)
[64]	254 nm	natural microbiome	1–2 m	PVC (in seawater)	no biofilm after 2 months continuous UV irradiation; UV reduced existing biofilms
[56]	254 nm, up to 1.47 mW/cm^2^	natural microbiome	2 d–7 d, ≈10^6^ cells/cm^2^ after 7 d	glass	irradiation reduced biofilm formation (>99% less biofilm cells after 7 d); however, even 1.47 mW/cm^2^ did not completely stop biofilm formation for 7 d
[65]	254 nm, up to 2 mW/cm^2^	natural microbiome	24 d, 10^6^–10^7^ cells/cm^2^	quartz (in sea water)	antifouling impact starts for >10 µW/cm^2^; however, even 0.8 mW/cm^2^ did not prevent biofilm formation completely
[58]	254 nm, up to 0.350 mW/cm^2^	*E. coli*	2 d	glass (in drinking water)	95% less biofilm volume @ 50.5 µW/cm^2^
[59]	254 nm, up to ≈0.15 mW/cm^2^	*E. coli*	2 d, 12 d, up to 27 µm	flow cell	0.06 mW/cm^2^ significantly reduced biofilm formation; however, biofilm formation even observed at 0.1 mW/cm^2^ and UVC is probably unable to stop biofilm formation in the long run (only 23 °C results)
[54]	265 nm	*P. aeruginosa*, *E. coli*		agar plate	4.3 mJ/cm^2^ to prevent biofilm (bacterial lawn) formation; (Irradiation via fibers)
[66]	265 nm, up to 21 mJ/cm^2^	*P. aeruginosa*	3 h	Teflon tubes	100% @ 1 mJ/cm^2^ (Teflon); no bacteria observed for 3–4 d; higher doses necessary for other materials (high NaCl concentration (20%) for light guide approach)
[48]	265 nm, 275 nm, 300 nm, 365 nm, up to 0.156 mW/cm^2^ (pulsed or continous)	mixture: *P. aeruginosa*, *Ralstonia insidiosa*, *Burkholderia multivorans*, *Cupriavidus metallidurans*, *Methylobacterium fujisawaense*	up to 6 d, ≈0.3 mm; 6.2 × 10^6^ cells/cm^2^	steel	265/275 nm: significant biofilm prevention at about 10 µW/cm^2^ (continuous/pulsed) at least for 6 days; 300/365 nm: no biofilm prevention but biofilm increase (irradiation via optical fibers; no total biofilm prevention even above 10 µW/cm^2^)
[57]	267 nm, 1 mW/cm^2^; up to 60 mJ/cm^2^	*C. auris*	24 h	steel, PS, poly-cotton	5–60 mJ/cm^2^ needed for a significant reduction in biofilm formation, depending on surface structure
[67]	272 nm, up to 0.48 mW/cm^2^ (pulsed or continuous)	natural microbiome	up to 24 w	quartz (in sea water)	almost no biofilm after 69 d @ 0.48 mW/cm^2^
[52]	273 nm, <0.2 mW/cm^2^	natural microbiome	up to 19 w	seachest with antifouling coating (in sea water)	UV-irradiation prevented/delayed biofilm formation
[49]	275 nm, up to 0.25 mW/cm^2^ (pulsed or continuous)	*P. aeruginosa*	up to 3 d, ≈250 µm	steel	significant biofilm prevention at about 8 µW/cm^2^ (irradiation via optical fibers; no total biofilm prevention even above 8 µW/cm^2^)
[51]	278 nm, 0.0174 mW/cm^2^	natural microbiome	up to 47 d	plastic (in sea water)	biofilm prevented for 47 d
[53]	278 nm	natural microbiome	up to 10 m	silicone (in sea water)	9 cm disk quite biofilm-free after 4 weeks in water with an average irradiation of 0.005 mW/cm^2^
[68]	280 nm, up to 0.093 mW/cm^2^; up to 167 mJ/cm^2^	natural microbiome	9 m	quartz	UV reduced biofilm formation; even 0.0005 µW/cm^2^ seemed to have an impact
[69]	UVC LED, ≈0.1 mW/cm^2^	natural microbiome	20 d	glass/polymer	biofilm CFU 1.8 log lower compared to unirradiated control after 20 d
[70]	281 nm, up to 0.108 mW/cm^2^; up to 18,700 mJ/cm^2^	*Navicula incerta*	up to 5 d, ≈10^5^ algae/cm^2^	tiles	1 log-reduction (biofilm cell) @ 42,000 mJ/cm^2^, 3 log-reduction @ 5 d and 5.77 µW (2500 mJ/cm^2^)
[71]	285 nm	natural microbiome	1 w–19 w	quartz (in sea water)	UV reduced biofilm formation
[72]	285 nm, 0.025 mW/cm^2^ up to 180 J/cm^2^	natural microbiome	112 d	quartz (in sea water)	irradiation delayed biofilm formation
[60]	385 nm, 420 nm, 2.5 mW/cm^2^; 216 J/cm^2^	*E. coli*	up to 24 h	silicone (in urine mucine medium in MTP)	2.5 mW/cm^2^ (216 J/cm^2^) reduced bacteria on silicone/medium and prevented biofilm formation
[73]	broadband blue (380–440 nm with peak @ 405 nm), 30.9 mW/cm^2^; 9.26 J/cm^2^	*S. mutans*	12–16 h	PS in medium in MTP	irradiation reduced biofilm formation (biofilm recovered for 2–6 h before analysis; tryptic soy broth might contain photosensitizer?)
[62]	405 nm, up to 160 mW/cm^2^ up to 1728 J/cm^2^	*Proteus mirabilis*		silicone (in artificial urine)	1 log-reduction in biofilm formation @ 18–32 mW/cm^2^ (194–346 J/cm^2^); 1.7 log-reduction @ 30–50 mW/cm^2^ (324–540 J/cm^2^); total biofilm prevention @ 160 mW/cm^2^ (1,728 J/cm^2^)
[74]	405 nm, 26 mW/cm^2^; up to 748.8 J/cm^2^	*L. monocytogenes*	24 h	steel and acryl in salmon exudate	irradiation reduced biofilm formation by ≈1 log @ 26 mW/cm^2^ or 748.8 J/cm^2^ (irradiation impact slightly temperature dependent)
[75]	410 nm, 455 nm, 100 mW/cm^2^; up to 450 J/cm^2^	*P. aeruginosa*	6 h	PS MTP	biofilm formation prevention: 410 nm: 6.6 log @ 450 J/cm^2^; 450 nm: 3.8 log @ 450 J/cm^2^;
[76]	445 nm (laser), 970 nm (laser), different irradiances; up to 120 J/cm^2^	*P. aeruginosa*	24 h, 72 h	MTP, flow cell, wound	one time 445 nm irradiation inhibited growth up to 18 h, but had mostly no larger effect after 24 h besides a small biomass reduction; no effect by 970 nm irradiation;
[77]	450 nm (pulsed), 2 mW/cm^2^; 7.6 J/cm^2^ three times per day over three days (68.4 J/cm^2^ total)	*S. aureus*, *P. acnes*	3 d	PS MTP	no significant impact on forming biofilms for the first three days
[78]	450 nm, 525 nm, 625 nm, up to 240 J/cm^2^	*C. albicans*	24 h	MTP	450 nm irradiation led to an average reduction of up to 0.43 log @ 240 J/cm^2^; no effects for other wavelengths;
[79]	“blue”, up to 1300 lux	*E. coli*	24 h	MTP	blue light reduced biofilm formation

### 3.3. Biofilm Irradiation for Biofilm Reduction

Table 4 presents the key data of almost 90 papers, which report the continuous or pulsed irradiation of existing biofilms in the spectral range 220–1000 nm. Most of the biofilms were mono-species biofilms cultured for one to three days. The most frequently examined microorganisms were the following bacteria: *P. aeruginosa* (Gram-negative), *S. aureus* (Gram-positive), *E. coli* (Gram-negative) and *L. monocytogenes* (Gram-positive). There are far fewer studies on fungal biofilms. Here, biofilms of *C. albicans* have been studied most frequently. The background of most of the investigations were medical issues or biofilm problems in the food or water sector.

**Figure 1:**
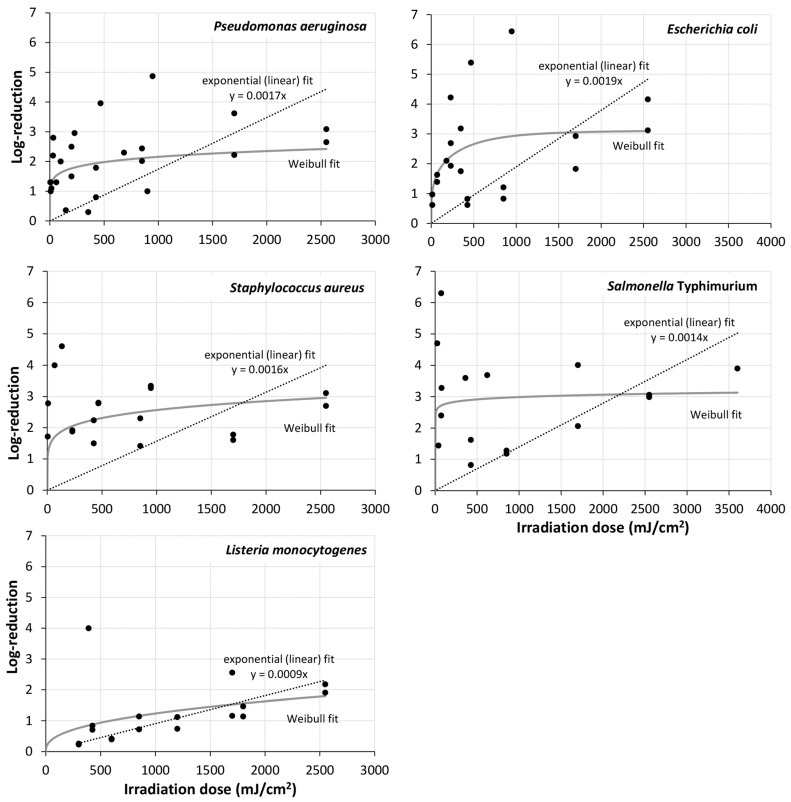
Log-reductions of *Pseudomonas* *aeruginosa*, *Escherichia coli*, *Staphylococcus aureus*, *Salmonella* Typhimurium and *Listeria monocytogenes* in mono-species biofilms, as affected by UVC irradiation. The analysis is based on the values displayed in Table 4. Dotted line: exponential linear fit, unbroken line: Weibull fit.

**Figure 2:**
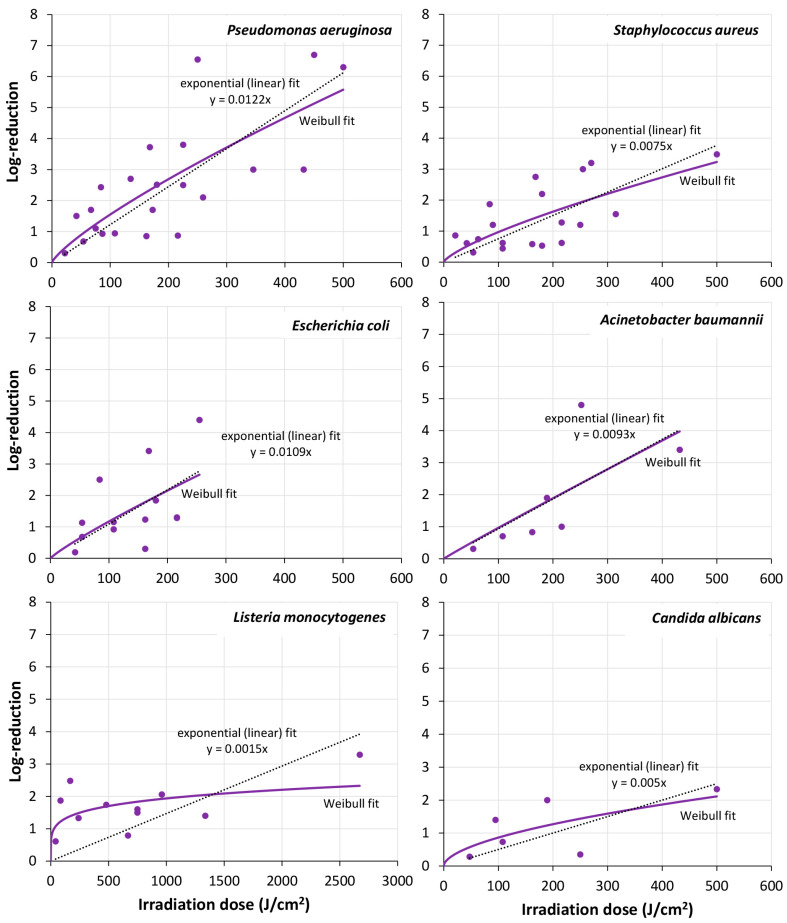
Log-reductions of *Pseudomonas aeruginosa*, *Staphylococcus aureus*, *Escherichia coli*, *Acinetobacter baumannii*, *Listeria monocytogenes* and *Candida albicans* in mono-species biofilms, as affected by visible violet light (400–420 nm) irradiation. The analysis is based on the values displayed in Table 4. Dotted line: exponential linear fit, purple unbroken line: Weibull fit. NB! The *x*-axis displayed for *Listeria monocytogenes* deviates from the ones in all other sub-figures.

**Figure 3:**
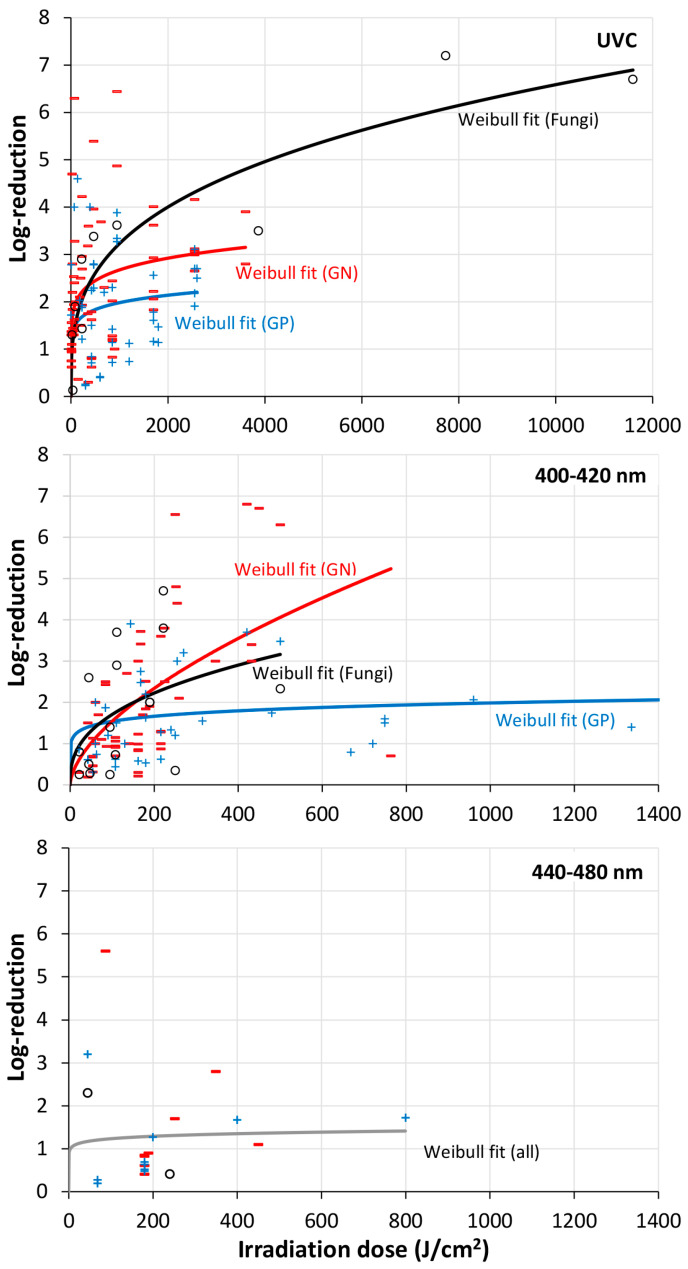
Overall cell reduction in mono-species biofilms formed by Gram-positive (GP, blue cross) and Gram-negative (GN, red bars) as well as fungi (black circles) by irradiation with UVC, visible violet (400–420 nm) as well as blue (440–480 nm) light. The analysis is based on the values displayed in Table 4. For data on UVC and violet light, unbroken blue, red, and black lines indicate Weibull fit for GP- and GN-bacteria as well as fungi, respectively. The unbroken gray line displayed for blue light represents the overall Weibull fit for all three groups. NB! The *x*-axis displayed for UVC deviates from the ones in all other sub-figures.

There were not many investigations in the UVB and UVA range. Most irradiations were performed with UVC radiation or visible violet/blue light. The result of the single microorganisms/mono-species biofilms in the UVC (200–280 nm) and visible violet spectral range (400–420 nm) are presented in Figure 1 and Figure 2, while Figure 3 offers an overview of all results of all irradiated mono-species biofilm in the UVC, violet region, and blue spectral region, divided in three subfigures.

**Figure 4:**
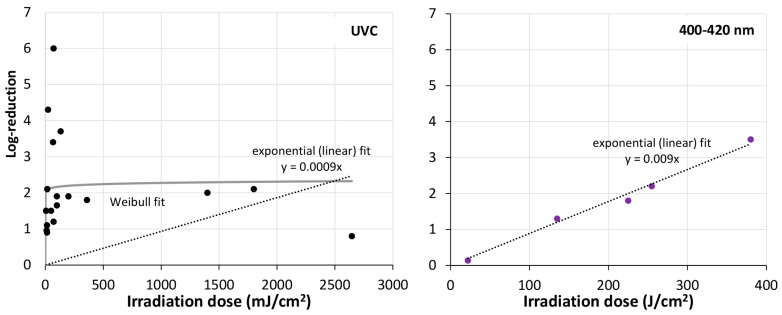
Overall cell reduction in multi-species biofilms by irradiation with UVC and visible violet (400–420 nm) light. The analysis is based on the values displayed in Table 3. Exponential (linear) fits (dotted line) are displayed for both exposure to UVC and visible violet light; Weibull fit (gray unbroken line) is only shown for multi-species biofilms exposed to UVC. NB! The *x*-axis displayed for UVC and visible violet light deviate from each other.

A total of five investigations dealt with natural biofilms. However, they were very difficult to compare. Three of them were grown in (sea) water and two on patient material. One was irradiated by blue light, the others irradiated by UVC. The maximum UVC irradiation doses differed by a factor of 500,000, nevertheless resulting in more or less similar log-reductions. An overview of the impact of irradiation in the UVC and visible spectral range on natural biofilms and artificial multi-species biofilms can be found in Figure 4.

Many authors compared the irradiation sensitivity of planktonic cells and cells in biofilms. In most reports cells in biofilms were more or much more resistant to irradiation compared to planktonic cells [77,80,81,82,83,84,85,86,87,88,89,90,91,92,93,94]. Only in three papers no difference between planktonic cells and cells in biofilms were observed or the sensitivity of the biofilm cells were even higher [95,96,97]. The reasons for these contradictory observations are unknown but may be caused by the differences in the experimental setup and procedure.

The publications reporting on irradiation with pulsed broadband xenon lamps were not evaluated here as they were even more difficult to compare. The applied lamps seem to have different emission spectra and maybe even additional different physical properties like pulse length. Unfortunately, the irradiation doses were given in many different units, including Farad, which is the unit of electrical capacitance and cannot be converted into irradiation units.

**Table 4:** Irradiation of biofilms. (“red” background: no biofilm-reducing effect of irradiation—“green” background: biofilm-reducing effect of irradiation; PC: polycarbonate, PE: polyethylene, PET: polyethylene terephthalate, PMMA: polymethyl methacrylate, PS: polystyrene, PTFE: polytetra fluoroethylene, PVC: polyvinylchloride, MTP: microtiter plate).

Reference	Irradiation Wavelength, Irradiance, Dose	Microorganisms	Biofilm Age, Thickness, Cells	Biofilm Substrate	Reduction
[98]	222 nm, up to 0.6 mW/cm^2^; up to 179.3 mJ/cm^2^	*E. coli*, *S. epidermis*	5 h, ≈10^6^ cells/cm^2^	PS MTP	*E. coli*: 2.10 log @ 179.3 mJ/cm^2^, S. epidermis: 2.03 log @ 179.3 mJ/cm^2^
[99]	222 nm, 254 nm, up to 600 mJ/cm^2^	*F. nucleatum*, *P. gingivalis*	72 h, 25 µm, 38 µm	plastic MTP	reduction in biofilm thickness: 222 nm: *F. nucleatum* and *P. gingivalis*; 254 nm: *F. nucleatum*
[87]	222 nm, 254 nm, 260 nm, 270 nm, 282 nm	*P. aeruginosa*	1 d–5 d	PC, quartz	≈1 log @ 55 mJ/cm^2^, 222 nm, 72 h ≈1 log @ 8.2 mJ/cm^2^, 270 nm, 72 h
[100]	249–338 nm in 5 nm steps (UVC, UVB, UVA), up to 2110 mJ/cm^2^	*P. aeruginosa*	24 h, 48 h, ≈100 µm (48 h)	cellulose nitrate membrane filter	for 24 h biofilm @126–170 mJ/cm^2^: UVC: 0.36 log; UVB (296 nm): up to 2.4 log @ 296 nm; UVA: no significant reduction; 48 h biofilm much more resistant;
[101]	254 nm	*L. monocytogenes*	7 d	steel	cells in biofilm reduced
[102]	254 nm, up to 1800 mJ/cm^2^	*L. monocytogenes*	24 h	steel, egg shell	steel: 0.26 log @ 300 mJ/cm^2^; 0.42 log @ 600 mJ/cm^2^; 1.12 log @ 1200 mJ/cm^2^; 1.47 log @ 1800 mJ/cm^2^; egg shell: 0.23 log @ 300 mJ/cm^2^; 0.40 log @ 600 mJ/cm^2^; 0.74 log @ 1200 mJ/cm^2^; 1.14 log @ 1800 mJ/cm^2^;
[103]	254 nm, 1.3 mW/cm^2^; up to 390 mJ/cm^2^	*L. monocytogenes*	24 h, ≈10^6^ cells/cm^2^	lettuce, cabbage	cell reduction in biofilm on both surfaces: » 4.0 log @ 390 mJ/cm^2^
[104]	254 nm	*L. monocytogenes*	6 d, 12 d, ≈10^6^ cells/cm^2^ (12 d)	steel	≥5 log cell reduction in biofilm
[105]	254 nm, up to 60 mJ/cm^2^	*V. parahaemolyticus*	24 h, ≈10^7^ cells/cm^2^	shrimp, crab	shrimp: 1.37 log @ 5 mJ/cm^2^; 1.56 log @ 10 mJ/cm^2^; 1.84 log @ 30 mJ/cm^2^; 2.53 log @ 60 mJ/cm^2^; crab: 0.75 log @ 5 mJ/cm^2^; 0.94 log @ 10 mJ/cm^2^; 1.37 log @ 30 mJ/cm^2^; 1.94 log @ 60 mJ/cm^2^;
[106]	254 nm, 0.236 mW/cm^2^; up to 2549 mJ/cm^2^	*P. aeruginosa*, *S. aureus*, *E. coli*, *L. monocytogenes*, S. Typhimurium	24 h	biofilms from agar transferred to steel, PP	steel: *P. aeruginosa*: 0.80 log @ 425 mJ/cm^2^; 2.02 log @ 850 mJ/cm^2^; 2.22 log @ 1700 mJ/cm^2^; 2.65 log @ 2549 mJ/cm^2^; *S. aureus*: 2.24 log @ 425 mJ/cm^2^; 1.42 log @ 850 mJ/cm^2^; 1.61 log @ 1700 mJ/cm^2^; 2.70 log @ 2549 mJ/cm^2^; *E. coli*: 0.62 @ 425 mJ/cm^2^; 0.83 log @ 850 mJ/cm^2^; 1.83 log @ 1700 mJ/cm^2^ 3.12 log @ 2549 mJ/cm^2^; *L. monocytogenes*: 0.84 @ 425 mJ/cm^2^; 1.14 log @ 850 mJ/cm^2^; 2.56 log @ 1700 mJ/cm^2^; 2.18 log @ 2549 mJ/cm^2^; *S.* Typhimurium: 0.82 log @ 425 mJ/cm^2^; 1.28 log @ 850 mJ/cm^2^; 2.06 log @ 1700 mJ/cm^2^; 3.06 log @ 2549 mJ/cm^2^; polypropylene: *P. aeruginosa*: 1.79 log @ 425 mJ/cm^2^; 2.44 log @ 850 mJ/cm^2^; 3.62 log @ 1700 mJ/cm^2^; 3.09 log @ 2549 mJ/cm^2^; *S. aureus:* 1.50 log @ 425 mJ/cm^2^; 2.30 log @ 850 mJ/cm^2^; 1.78 log @ 1700 mJ/cm^2^; 3.11 log @ 2549 mJ/cm^2^; *E. coli*: 0.82 @ 425 mJ/cm^2^; 1.21 log @ 850 mJ/cm^2^; 2.93 log @ 1700 mJ/cm^2^; 4.16 log @ 2549 mJ/cm^2^; *L. monocytogenes*: 0.71 @ 425 mJ/cm^2^; 0.72 log @ 850 mJ/cm^2^; 1.16 log @ 1700 mJ/cm^2^; 1.91 log @ 2549 mJ/cm^2^; *S*. Typhimurium: 1.62 log @ 425 mJ/cm^2^; 1.18 log @ 850 mJ/cm^2^; 4.01 log @ 1700 mJ/cm^2^; 2.99 log @ 2549 mJ/cm^2^;
[89]	254 nm (irradiation from top or bottom for up to 60 min), up to 0.63 mW/cm^2^; up to 1400 mJ/cm^2^	*P. aeruginosa*	4 d	on quartz Petri dish	0.3 log @ ≈354 mJ/cm^2^; 1 log @ ≈900 mJ/cm^2^; 100% @ 1300 mJ/cm^2^; (“inside out” irradiation more effective; planktonic cells more sensitive than cells in biofilm)
[82]	254 nm, up to 40 mJ/cm^2^	*C. neoformans*	up to 48 h	PS	0.13 log @ 40 mJ/cm^2^; (planktonic cells more sensitive than cells in biofilm)
[91]	254 nm	*F. solani*	up to 48 h	PS MTP	cells in biofilm are reduced (planktonic cells more sensitive than cells in biofilm)
[107]	254 nm	*P. aeruginosa*, *S. aureus*, *S. epidermis,* *A. baumannii,* *E. coli*	24 h	MTP	strong cell reduction in all biofilms, (no large change in biomass)
[94]	254 nm	*P. aeruginosa*, *E. coli*, *S. aureus* MSSA, *S. aureus* MRSA, *S. epidermis* MRSE, *C. albicans*	24 h	steel	*P. aeruginosa*: 2.96 log @ 228.6 mJ/cm^2^; 3.96 log @ 467.8 mJ/cm^2^; 4.87 log @ 946.7 mJ/cm^2^; *E. coli*: 4.22 log @ 228.6 mJ/cm^2^; 5.39 log @ 467.8 mJ/cm^2^; 6.44 log @ 946.7 mJ/cm^2^; *S. aureus* (MSSA): 1.88 log @ 228.6 mJ/cm^2^; 2.78 log @ 467.8 mJ/cm^2^; 3.34 log @ 946.7 mJ/cm^2^; *S. aureus* (MRSA): 1.92 log @ 228.6 mJ/cm^2^; 2.80 log @ 467.8 mJ/cm^2^; 3.27 log @ 946.7 mJ/cm^2^; *S. epidermis*: 1.21 log @ 228.6 mJ/cm^2^; 2.29 log @ 467.8 mJ/cm^2^; 3.88 log @ 946.7 mJ/cm^2^; *C. albicans*: 1.43 log @ 228.6 mJ/cm^2^; 3.38 log @ 467.8 mJ/cm^2^; 3.62 log @ 946.7 mJ/cm^2^;
[83]	254 nm, 1.4 mW/cm^2^; up to 2600 mJ/cm^2^	*A. acidoterrestris,* *A. herbarius,* *A. cycloheptanicus,* *A. acidocaldarius*	72 h	steel, rubber	steel: 2.5 log @ 2600 mJ/cm^2^; rubber: 2.7 log @ 2600 mJ/cm^2^ (planktonic spores much more sensitive than cells in biofilm)
[56]	254 nm, up to 1.47 mW/cm^2^	natural microbiome	2 d, ≈5 × 10^5^ cells/cm^2^	glass	84%/0.8 log cell reduction in 2 d biofilm @ 2646 mJ/cm^2^;
[108]	254 nm, up to 6,000,000 mJ/cm^2^	natural microbiome	>100 d, ≈10^4^ cells/cm^2^	steel (in ground water)	≈1.6 CFU log-reduction @ 6,000,000 mJ/cm^2^
[109]	254 nm, 0.4 mW/cm^2^; up to 2160 mJ/cm^2^	natural patient biofilm	mature	silicone urinary catheter	≈0.96 log @ 12 mJ/cm^2^; ≈2 log @ 1400 mJ/cm^2^; (planktonic cells more sensitive than cells in biofilm)
[110]	254 nm, 0.7 mW/cm^2^; up to 210 mJ/cm^2^	*C. albicans*	24 h	PMMA	1.3 log @ 21 mJ/cm^2^; 1.9 log @ 84 mJ/cm^2^; 2.9 log @210 mJ/cm^2^;
[111]	254 nm, 6.4 mW/cm^2^; 1920 mJ/cm^2^	*S. aureus,* *S. epidermis*	24 h	plastic	reduction below ≈5% (irradiation details unclear)
[112]	254 nm, up to 620 mJ/cm^2^	*S.* Typhimurium	48 h, 3 × 10^6^ cells/cm^2^	steel	1.44 log @ 39.5 mJ/cm^2^; 3.28 log @ 76.4 mJ/cm^2^; 3.69 log @ 620.4 mJ/cm^2^;
[113]	254 nm, 1.2 mW/cm^2^; up to 360 mJ/cm^2^	*S*. Typhimurium, cultivable indigenous microorganisms (CIM)	72 h (steel) ≈10^7^ cells/cm^2^, 24 h (lettuce) ≈3 × 10^4^–7 × 10^6^ cells/cm^2^	steel, lettuce	steel: *S*. Typhimurium: 4.7 log @ 24 mJ/cm^2^; 6.3 log @ 72 mJ/cm^2^; *S*. Typhimurium mixed: 4.3 log @ 24 mJ/cm^2^; 6.0 log @ 72 mJ/cm^2^; lettuce: *S*. Typhimurium: 2.4 log @ 72 mJ/cm^2^; 3.6 log @ 360 mJ/cm^2^; *S*. Typhimurium mixed: 1.2 log @ 72 mJ/cm^2^; 1.8 log @ 360 mJ/cm^2^; (multi-species biofilms less sensitive)
[114]	254 nm, 3.5 mW/cm^2^	*C. auris*	48 h	PS	3.5 log @ 3864 mJ/cm^2^; 7.2 log @ 7728 mJ/cm^2^; 6.7 log @ 11,592 mJ/cm^2^;
[115]	UVC LED (254 nm?), irradiation up to 20 min	mixture: *S. mutans,* *S. aureus,* *E. coli,* *C. albicans*	24 h	silicone	significant biofilm reduction for 20 min UVC
[116]	254 nm, 3.1 mW/cm^2^, up to 11,160 mJ/cm^2^	*Navicula incerta*	60 min	glass	biofilm reduction
[117]	254 nm, 0.625 mW/cm^2^, up to 200 mJ/cm^2^; 270 nm, 0.038 mW/cm^2^, up to 100 mJ/cm^2^; 405 nm, 75.5 mW/cm^2^, up to 225 J/cm^2^	*P. aeruginosa,* natural microbiome	3 d, *P. aeruginosa*: 1.8 × 10^8^ CFU/cm^2^; mixed culture: 1.4 × 10^5^ CFU/cm^2^	PC, PTFE, PVC, quartz	*P. aeruginosa* biofilm on PC: 254 nm: 1.1 log @ 15 mJ/cm^2^ 1.3 log @ 60 mJ/cm^2^ 1.5 log @ 200 mJ/cm^2^ 270 nm: 1.3 log @ 4.5 mJ/cm^2^ 2.2 log @ 30 mJ/cm^2^ 2.0 log @ 100 mJ/cm^2^ 2.5 log @ 200 mJ/cm^2^ 405 nm: 0.3 log @ 22 J/cm^2^ 1.7 log @ 67 J/cm^2^ 2.7 log @ 135 J/cm^2^ 3.8 log @ 225 J/cm^2^ dual species biofilm on PC: 254 nm: 1.1 log @ 15 mJ/cm^2^ 1.65 log @ 100 mJ/cm^2^ 1.9 log @ 200 mJ/cm^2^ 270 nm: 0.9 log @ 15 mJ/cm^2^ 1.5 log @ 50 mJ/cm^2^ 1.9 log @ 100 mJ/cm^2^ 405 nm: 0.14 log @ 22 J/cm^2^ 1.3 log @ 135 J/cm^2^ 1.8 log @ 225 J/cm^2^
[118]	255 nm, 0.088 mW/cm^2^; up to 135 mJ/cm^2^	*S. aureus*, *A. baumannii*		PVC	*S. aureus*: 1.72 log @ 3.7 mJ/cm^2^; 2.78 log @ 7.4 mJ/cm^2^; 4.0 log @ 66.7 mJ/cm^2^; 4.6 log @ 133 mJ/cm^2^; *A. baumanni*: 0.34 log @ 5.0 mJ/cm^2^; 0.92 log @ 17.4 mJ/cm^2^; 1.5 log @ 66.7 mJ/cm^2^; 1.5 log @ 133 mJ/cm^2^; dual species: 1.5 log @ 7.4 mJ/cm^2^; 2.1 log @ 17.4 mJ/cm^2^; 3.4 log @ 66.7 mJ/cm^2^; 3.7 log @ 133 mJ/cm^2^;
[119]	265 nm, up to 1570 mJ/cm^2^	*P. aeruginosa*	3 d	Teflon and silicone urinary catheter	≈4 log @ 7.9 mJ/cm^2^ (high NaCl concentrations of up to 20% to achieve light guide effect ⇒ therefore values not included in analysis)
[120]	265 nm	*P. aeruginosa*	48 h	PC	≈1.3 log @ 8 mJ/cm^2^ ≈2.8 log @ 32 mJ/cm^2^
[121]	265 nm, 1.93 mW/cm^2^; up to 231.6 mJ/cm^2^	*P. aeruginosa*	48 h	chamber well slides	irradiation led to dead biomass; no increase in dead biomass after about 13 mJ/cm^2^
[122]	266 nm (UVC), up to 1000 mJ/cm^2^; 296 nm (UVB), up to 2000 mJ/cm^2^;	*P. aeruginosa*	24 h, 48 h, 72 h, ≈200 µm	cellulose nitrate membrane filter	UVC: ≈1 log @ 1000 mJ/cm^2^ (24 h) UVB: ≈1 log @ 63.8 mJ/cm^2^ (24 h) ≈4.1 log @ 200 mJ/cm^2^ (24 h); 48 h and 72 h biofilm more resistant
[93]	268 nm (UVC) 275 nm (UVC) 312 nm (UVB) 370 nm (UVA)	*E. coli*	24 h, ≈431 nm	PES membrane	268 nm: 0.62 log @ 12 mJ/cm^2^; 1.39 log @ 69 mJ/cm^2^; 1.93 log @ 230 mJ/cm^2^; 1.75 log @ 347 mJ/cm^2^; 275 nm: 0.97 log @ 12 mJ/cm^2^; 1.63 log @ 69 mJ/cm^2^; 2.69 log @ 230 mJ/cm^2^; 3.18 log @ 347 mJ/cm^2^; 312 nm: 0.66 log @ 23 mJ/cm^2^; 0.95 log @ 69 mJ/cm^2^; 1.17 log @ 150 mJ/cm^2^; 1.25 log @ 230 mJ/cm^2^; 370 nm: 0.02 log @ 23 mJ/cm^2^; 0.38 log @ 69 mJ/cm^2^; 1.17 log @ 150 mJ/cm^2^; 1.25 log @ 230 mJ/cm^2^;
[123]	275 nm (pulsed), 6 mW/cm^2^; 455 nm (pulsed), 291 mW/cm^2^	*S.* Typhimurium, *A. australiensis*	up to 6 d, ≥10^7^ cells/cm^2^ depending on biofilm and time	steel	*S*. Typhimurium: 275 nm: 3.9 log @ 3600 mJ/cm^2^; 455 nm: 2.8 log @ 349.2 J/cm^2^; *A. australiensis*: 275 nm: 2.8 log @ 3600 mJ/cm^2^; 455 nm: 5.6 log @ 87.3 J/cm^2^; dual species: 275 nm: 2.1 log @ 1800 mJ/cm^2^; 455 nm: 4.3 log @ 87.3 J/cm^2^;
[124]	280 nm, 0.57 mW/cm^2^; up to 684 mJ/cm^2^	*P. aeruginosa*, *L. citreum*	24 h, 10^8^–10^9^ cells/cm^2^	cellulose ester membranes	*P. aeruginosa*: 2.3 log @ 684 mJ/cm^2^; *L. citreum*: 2.2 log @ 684 mJ/cm^2^;
[72]	285 nm, 0.025 µW/cm^2^; up to 180 mJ/cm^2^ (one time irradiation)	natural microbiome	14 d	quartz	irradiation reduced further biofilm growth
[125]	365 nm, 2.5 mW/cm^2^; up to 216 J/cm^2^	*P. aeruginosa*	0.5 h, 1 h, 24 h, ≥10^8^ cells/cm^2^	glass	UVA irradiation slightly promoted biofilm formation
[92]	365 nm, 2 mW/cm^2^; up to 21.6 J/cm^2^	*P. aeruginosa*	24 h	glass	≈1.5 log @ 21.6 J/cm^2^
[126]	365 nm pulsed and CW, 0.28 mW/cm^2^; 1008 mJ/cm^2^	*E. coli*, *C. albicans*	*E. coli*: 48 h; *C. albicans*: 72 h;	MTP	*E. coli*: 3.4 log @ 1008 J/cm^2^; *C. albicans*: 3.1 log @ 1008 J/cm^2^; (100 Hz more effective than cw)
[60]	385 nm, 420 nm, 2.5 mW/cm^2^; 216 J/cm^2^;	*E. coli*	up to 24 h	silicone (in urine mucine medium in MTP)	24 h biofilms: in urine mucin medium: no reduction @ 216 J/cm^2^ for both wavelengths; in PBS: 2.2 log @ 216 J/cm^2^ of 385 nm; 1.3 log @ 216 J/cm^2^ of 405 nm;
[80]	400 nm, 60 mW/cm^2^; up to 216 J/cm^2^	*P. aeruginosa,* *S. aureus*, *E. coli*, *A. baumannii*, amongst others	72 h	PP	@ 54/108/162/216 J/cm^2^: *P. aeruginosa*: 0.68/0.94/0.85/0.87; *S. aureus*: 0.32/0.44/0.58/0.63; *E. coli*: 1.13/1.15/1.23/1.28; *A. baumannii*: 0.31/0.7/0.83/1.0; (planktonic cells more sensitive than cells in biofilm)
[127]	400 nm, 420 nm, 570 nm, 583 nm, 698 nm, up to 29.2 mW/cm^2^; up to 420.5 J/cm^2^	*P. fluorescens*, *S. epidermis*	24 h, *P. fluorescens* ≈10^8^ cells/cm^2^; *S. epidermis* ≈10^7^ cells/cm^2^	PS	*P. fluorescens* @ 400 nm: 1 log @ ≈140 J/cm^2^, 29.1 mW/cm^2^; 6.8 log @ ≈420.5 J/cm^2^, 29.1 mW/cm^2^; less strong reduction at 420 nm, no reduction at other wavelengths; *S. epidermis* @ 400 nm: 1 log @ ≈130 J/cm^2^, 29.1 mW/cm^2^; 3.7 log @ ≈420.5 J/cm^2^, 29.1 mW/cm^2^; no reduction at other wavelengths
[128]	405 nm (laser), 300 mW/cm^2^; up to 270 J/cm^2^	*S. aureus*	3 d	urethral stent in broth	1.2 log @ 90 J/cm^2^; 2.2 log @ 180 J/cm^2^; 3.2 log @ 270 J/cm^2^;
[88]	400 nm: up to 99.7 J/cm^2^; 470 nm: up to 306.3 J/cm^2^, 522 nm, 644 nm	*P. fluorescens*	24 h, 10^7^–10^8^ cells/cm^2^	PS (hydrated)	no significant changes in biofilm (planktonic cells (more) sensitive to violet light)
[129]	402 nm, 440 nm, 35 mW/cm^2^; up to 252 J/cm^2^	*A. baumannii*	24 h	MTP	402 nm: 1.9 @ 189 J/cm^2^; 4.8 log @ 252 J/cm^2^; 440 nm: 0.9 log @ 189 J/cm^2^; 1.7 log @ 252 J/cm^2^;
[130]	403 nm laser, 141 mW/cm^2^; up to 21.16 J/cm^2^	*S. aureus*	8 h–48 h	MTP	24 h biofilm: 0.86 log @ 21.2 J/cm^2^ 48 h biofilm: 0.26 log @ 21.2 J/cm^2^
[131]	405 nm: 84 mW/cm^2^; 379–452 nm: 62 mW/cm^2^;	*P. aeruginosa,* *S. aureus*, *E. coli,* *A. baumannii*	72 h	PP	average log-reduction @ 513 J/cm^2^ of 405 nm (“SWA”): *P. aeruginosa* 0.64; *S. aureus* 0.4; *E. coli* 0.97; *A. baumannii* 0.63; 395 nm exhibits similar antimicrobial impact; other wavelengths less antimicrobial; (not included in analysis because of seemingly inhomogeneous irradiation)
[132]	405 nm, 80 mW/cm^2^; 144 J/cm^2^	*P. acnes*	up to 7 d	PET membrane	3.9 log @ 144 J/cm^2^
[133]	405 nm, 60 mW/cm^2^; 216 J/cm^2^	*S. aureus*	48 h	MTP	0.62 log @ 108 J/cm^2^ 1.28 log @ 216 J/cm^2^
[134]	405 nm, 1050 mW/cm^2^;	*S. aureus*	48 h	titanium	0.74 log @ 63 J/cm^2^; 1.55 log @ 315 J/cm^2^;
[135]	405 nm, 150 mW/cm^2^; up to 3240 J/cm^2^;	*S. aureus*	72 h	skin/titanium	1.63 log @ 3240 J/cm^2^
[90]	405 nm, 60 mW/cm^2^, up to 216 J/cm^2^	*M. catarrhalis*	24 h	MTP	≈3.6 @ 216 J/cm^2^ (planktonic cells somewhat more light sensitive)
[74]	405 nm, 26 mW/cm^2^; up to 748.8 J/cm^2^	*L. monocytogenes*	24 h	steel and acryl in salmon exudate	@ 25 °C: steel: 1.5 log @ 748.8 J/cm^2^ acryl: 1.6 log @ 748.8 J/cm^2^
[96]	405 nm, 24 mW/cm^2^; up to 432 J/cm^2^	*P. aeruginosa*	24 h + 48 h	steel	@ 25 °C: 0.93 log @ 86.4 J/cm^2^; 1.7 log @ 172.8 J/cm^2^; 2.1 log @ 259.2 J/cm^2^; 3.0 log @ 345.6 J/cm^2^; (cells in biofilm less light resistant than planktonic cells)
[136]	405 nm, 60 mW/cm^2^; up to 108 J/cm^2^	*C. albicans*	48 h	MTP	0.73 log @ 108 J/cm^2^ planktonic cells more sensitive than cells in biofilm
[137]	405 nm, up to 92.6 mW/cm^2^; up to 500 J/cm^2^	*P. aeruginosa,* *S. aureus,* *C. albicans*	24 h, 48 h, 10^7^–10^8^ cells/cm^2^	MTP, PC	@ 24 h biofilm after 250/500 J/cm^2^: *P. aeruginosa*: 6.55/6.3 log *S. aureus*: 1.2/3.48 log *C. albicans*: 0.35/2.33 log; *P. aeruginosa* and *S. aureus*: *P. aeruginosa*: 3.94/3.4 log *S. aureus*: 1.42/2.37 log *P. aeruginosa* and *C. albicans*: *P. aeruginosa*: 5.67/6.34 log *C. albicans*: 2.46/3.11 @ 48 h MTP biofilm after 500 J/cm^2^: (biofilms grown on PC in CDC bioreactor slightly more resistant)
[138]	405 nm, 141.5 mW/cm^2^; up to 504 J/cm^2^	*P. aeruginosa*, *S. aureus*, *E. coli*, *L. monocytogenes*	4–72 h, glass: 10^6^–10^8^ cells/cm^2^; acrylic: 10^4^–10^5^ cells/cm^2^	glass, acryl	*P. aeruginosa* 24 h glass: 1.5 @ 42 J/cm^2^; 2.43 @ 84 J/cm^2^; 3.72 @ 168 J/cm^2^; *L. monocytogenes* 24 h glass: 0.61 @ 42 J/cm^2^; 1.87 @ 84 J/cm^2^; 2.48 @ 168 J/cm^2^; *E. coli* 24 h glass: 0.19 log @ 42 J/cm^2^; 2.5 log @ 84 J/cm^2^; 3.41 log 168 J/cm^2^; 4.4 log @ 254.7 J/cm^2^; *S. aureus* 24 h glass: 0.61 @ 42 J/cm^2^; 1.87 @ 84 J/cm^2^; 2.75 @ 168 J/cm^2^; 3.0 log @ 254.7 J/cm^2^; *E. coli* and *S. aureus* 24 h glass: 2.2 log @ 254.7 J/cm^2^; (mixed biofilm more resistant; biofilms became more resistant with maturity)
[139]	405 nm, 60 mW/cm^2^; up to 162 J/cm^2^	*E. coli*, *K. pneumoniae,* *K. oxytoca*	72 h	PP	@ 162/54/108 J/cm^2^: *E. coli*: 0.30/0.68/0.92; *K. pneumoniae*: 0.21/0.46/0.91; *K. oxytoca*: 0.99/0.69/1.06;
[140]	405 nm, 280 mW/cm^2^; up to 284.4 J/cm^2^	*C. albicans*, *C. glabrata*,	24 h	PMMA in artificial saliva	mono-species biofilms: *C. albicans* reduction: 0.28 log @ 47.4 J/cm^2^; 1.4 log @ 94.8 J/cm^2^; 2 log @ 189.6 J/cm^2^; *C. glabrata* reduction: 0.25 log @ 94.8 J/cm^2^; 2 log @ 189.6 J/cm^2^; no biofilm after 30 min (284 J/cm^2^) irradiation
[141]	405 nm, 280 mW/cm^2^; up to 379.7 J/cm^2^	*S. mutans*, *C. albicans*	24 h	PMMA in artificial saliva	dual-species biofilm: 3.64 log @ 189.6 J/cm*^2^* for *C. albicans* and 3.66 log @ 189.6 J/cm^2^ for *S. mutans* in dual species biofilm; faster reduction in *C. albicans* for higher doses;
[142]	405 nm, 280 mW/cm^2^; up to 379.7 J/cm^2^	*S. mutans*, *C. albicans*	24 h	PMMA in artificial saliva	mono-species biofilms: *S. mutans*: 3.6 log @ 379.7 J/cm^2^; *C. albicans*: 3.55 log @ 379.7 J/cm^2^; cell reduction in dual-species biofilms: *S. mutans*: 3.4 log @ 379.7 J/cm^2^; *C. albicans*: 3.57 log @ 379.7 J/cm^2^;
[143]	405 nm, 370.6 mW/cm^2^; up to 222 J/cm^2^	*B. bruxellensis*	30 d	steel, oak in wine or yeast medium	steel and yeast medium: 0.8 log @ 22 J/cm^2^; 2.6 log @ 44.5 J/cm^2^; 3.7 log @ 111 J/cm^2^; 3.8 log @ 222 J/cm^2^; wood and wine: 0.25 log @ 22 J/cm^2^; 0.5 log @ 44.5 J/cm^2^; 2.9 log @ 111 J/cm^2^; 4.7 log @ 222 J/cm^2^;
[97]	405 nm, up to 100 mW/cm^2^; up to 360 J/cm^2^	*V. vulnificus*	48 h MTP; 6 h wound	MTP, wounds	1 log @ ≈60 J/cm^2^; 3 log @ ≈162 J/cm^2^ (no large sensitivity differences between planktonic cells and cells in biofilms)
[144]	405 nm 420 nm 460 nm	*L. monocytogenes*	48 h, ≈6.5 µm	steel, PVC, silicone, PE, PS	steel: 405 nm: 0.79 log @ 668 J/cm^2^; 1.40 log @ 1336 J/cm^2^; 3.29 log @ 2672 J/cm^2^; 420 nm: 1.33 log @ 240 J/cm^2^; 1.74 log @ 480 J/cm^2^; 2.06 log @ 960 J/cm^2^; 460 nm: 1.27 log @ 200 J/cm^2^; 1.67 log @ 400 J/cm^2^; 1.72 log @ 800 J/cm^2^; significant biomass reduction for all wavelengths;
[75]	410 nm, 455 nm, 100 mW/cm^2^; up to 450 J/cm^2^	*P. aeruginosa*	6 h	PS MTP	410 nm: 1.1 log @ 75 J/cm^2^; 2.5 log @ 225 J/cm^2^; 6.7 log @ 450 J/cm^2^; 455 nm: 1.1 log @ 450 J/cm^2^
[145]	415 nm, up to 100 mW/cm^2^; up to 540 J/cm^2^	*P. aeruginosa,* *A. baumannii*	24 h, 72 h	MTP, wounds	MTP—*P. aeruginosa*: ≈3 log @ 432 J/cm^2^ for 24 and 72 h biofilm; MTP—*A. baumannii*: ≈3.6 and 3.2 log @ 432 J/cm^2^ for 24 and 72 h biofilm, respectively; wound—*A. baumannii*: ≈3 log @ 360–540 J/cm^2^
[146]	415 nm, 445 nm, 525 nm, 623 nm, up to 110 J/cm^2^	*P. aeruginosa*, *S. aureus*		plastic	415 nm: P*. aeruginosa* PAO1: ≥2 log @ 60 J/cm^2^ *P. aeruginosa* LESB65: ≥2 log @ 60 J/cm^2^ *S. aureus* CF-MRSA: ≥2 log @ 60 J/cm^2^ *S. aureus* USA300: ≈1 log@60 J/cm^2^, ≈1.5 log@110 J/cm^2^ 445 nm: *P. aeruginosa*: ≈1 log @ 60 J/cm^2^ *S. aureus*: ≈1 log @ 60 J/cm^2^ 525 nm: *P. aeruginosa* LESB65: ≈1 log @ 60 J/cm^2^; no reduction for other strains; 623 nm: no reduction
[86]	420 nm, 212 mW/cm^2^; up to 763 J/cm^2^	*P. fluorescens*	60 h	PS MTP in medium	≈0.7 log @ 763 J/cm^2^ (planktonic bacteria more light sensitive than bacteria in biofilms)
[147]	420 nm, 93 mW/cm^2^; 2 × 72 J/cm^2^ per day over 5 days (720 J/cm^2^ total)	*S. mutans*	5 d	saliva-coated hydroxyapatite	1 log @ 720 J/cm^2^ (in total); 42% biomass reduction;
[95]	420 nm, 455 nm, 480 nm, 50 mW/cm^2^; up to 180 J/cm^2^	*P. aeruginosa*, *S. aureus*, *S. epidermis,* *E. coli*	24 h	MTP in medium	420 nm @ 180 J/cm^2^ *P. aeruginosa*: 2.51; *S. aureus*: 0.53; *S. epidermis*: 1.63; *E. coli*: 1.84; 455 nm @ 180 J/cm^2^: *P. aeruginosa*: 0.83; *S. aureus*: 0.48; *S. epidermis*: 0.52; *E. coli*: 0.41; 480 nm @ 180 J/cm^2^: *P. aeruginosa*: 0.61; *S. aureus*: 0.69; *S. epidermis*: 0.63; *E. coli*: 0.85; (cells in biofilms more light sensitive than planktonic cells)
[76]	445 nm (laser), 380–490 nm (LED), 970 nm (laser), different irradiances; up to 120 J/cm^2^	*P. aeruginosa*	0.5 h, 24 h	MTP, wound	445 nm irradiation significantly reduced cells in 24 h biofilms in MTP with higher doses leading to a larger reduction; irradiated wound also exhibits reduced bacteria
[148]	450 nm, 57 mW/cm^2^; 100 J/cm^2^	*P. aeruginosa*	48 h	MTP	no significant biofilm reduction
[77]	450 nm (pulsed), 2 mW/cm^2^; 7.6 J/cm^2^ three times per day over three days (68.4 J/cm^2^ total)	*S. aureus*, *P. acnes*	24 h	PS MTP	MRSA: 0.276 log @ 68.4 J/cm^2^ (total); *P. acnes*: 0.194 log @ 68.4 J/cm^2^ (total); (cells in biofilms more light sensitive than planktonic cells)
[78]	450 nm, 525 nm, 625 nm, up to 240 J/cm^2^	*C. albicans*	24 h	MTP	450 nm: of 0.41 log @ 240 J/cm^2^; no antimicrobial effects for other wavelengths;
[149]	455 nm, 50 mW/cm^2^; 4 × 12 mJ/cm^2^	natural patient biofilm	3 d	MTP in medium	0.28 log @ 48 J/cm^2^ (biofilm microbiome constitution changed after irradiation)
[150]	455 nm, 75 mW/cm^2^; up to 45.2 J/cm^2^	*S. aureus*, *C. albicans*	14 d	bone	*S. aureus*: 3.2 log @ 45.2 J/cm^2^; *C. albicans*: 2.3 log @ 45.2 J/cm^2^
[151]	460 nm, red light, 60 mW/cm^2^; up to 240 J/cm^2^	*C. albicans*	24 h, 48 h, 72 h		460 nm reduced cells in biofilm; no visible impact of red light
[152]	390–480 nm (peak at 460 nm), 1000 mW/cm^2^; 60 J/cm^2^;	*E. faecalis*	3 w	teeth	0.05 log @ 60 J/cm^2^
[153]	blue light around 470 nm, 620 mW/cm^2^; up to 262 J/cm^2^	*S. mutans*	24 h, ≈85 µm	MTP in medium	biofilm regrowth increased after blue irradiation; however, bacterial viability decreased; blue light seemed to have a delayed antimicrobial impact
[81]	broadband blue (400–520 nm), 500 mW/cm^2^; up to 60 J/cm^2^	*A. actinomycetemcomitans,* *F. nucleatum*, *P. gingivalis*	7 d, up to 45 µm	MTP in medium	irradiation reduced mostly P*. gingivalis* cells in biofilm: 0.95 log @ 60 J/cm^2^; (planktonic cells much more light sensitive than cells in biofilm)
[154]	broadband blue (400–500 nm), 1140 mW/cm^2^; up to 68 J/cm^2^	*S. mutans*	24 h	MTP in medium	no effect on biofilm
[155]	400–500 nm, 1217 mW/cm^2^; 146 J/cm^2^;	*F. nucleatum*, *P. gingivalis,* *S. sanguinis*, *A. naeslundii*	48 h/72 h	hydroxyapatite in saliva	mono-species biofilms: *P. gingivalis* 0.2 log @146 J/cm^2^; no reduction for the other mono-species biofilms; (irradiation of the multi-species biofilm changed its bacterial composition)
[156]	broadband blue (400–500 nm), 1140 mW/cm^2^; up to 680 J/cm^2^;	*S. mutans*	24 h	MTP in medium	blue light seemed to have a delayed antimicrobial impact
[157]	broadband blue (400–500 nm), 623 mW/cm^2^; 112 J/cm^2^;	*S. mutans,* *S. sanguinis*	24 h, ≈200 µm	enamel (in PBS)	irradiation reduced viable cells in mono- and multi-species biofilm (biofilm recovered for 24–48 h before analysis)
[158]	pulsed (unknown spectrum)	*P. aeruginosa*	8 h, 48 h biofilms	MTP, PC membrane	up to 100% reduction @ unknown irradiation parameters; (mature biofilms more resistant)
[159]	pulsed Xenon (220–520 nm)	*P. aeruginosa,* *S. aureus,* *E. coli*	up to 72 h	PVC	reductions in several logs achieved
[160]	pulsed Xenon (200–1100 nm), 1270 mJ/pulse at a distance that was not applied against biofilms	*E. coli*, *L. monocytogenes*	24 h, 48 h	lettuce, PP	cell reduction in several logs in both bacteria; *E. coli* more sensitive than *L. monocytogenes*, mature biofilms more resistant; reduction slightly higher on polyethylene than on lettuce
[85]	pulsed Xenon (200–1000 nm)	*A. niger,* *P. glaucum*	8 h, 48 h	MTP, PC membrane	irradiation reduced cells in biofilm independent of biofilm maturity (planktonic much more sensitive than cells in biofilm)
[84]	pulsed Xenon (200–1000 nm), up to 40.7 mJ/cm^2^ per pulse; up to 21,978 J/cm^2^	*S. aureus,* *B. cereus,* *B. thuringiensis,* *L. moncytogenes,* *P. acidilacti,* *L. brevis*, *E. faecium*	8 h and 48 h	MTP, PC membrane	irradiation reduced cells in biofilm; more mature biofilm more resistant (planktonic cell more sensitive than cells in biofilm)
[161]	pulsed Xenon (220–520 nm), 16.2 J/pulse	*C. albican,* *C. parapsilosis*	48 h, 72 h	steel, PVC	3–4 log @ 6.48 µJ/cm^2^ (irradiation dose correct?)

For analyzing the observed data, we tried to find a simple fit function that describes the result best. A frequently applied approach is the assumption of an exponential decrease in the number of surviving cells for increased irradiation doses, called Chick-Watson [162,163] or Bigelow model [13] or “log-lin”, as in a half logarithmic representation the resulting curve would be a straight line. This seems to be more or less in agreement with at least some biofilm irradiation studies [37,90,97,110]. Several authors have observed deviations from these model [55,87,93,117,120,122,124,127]. A potential reason for this discrepancy could be the fact that cells in deeper biofilm layers are less irradiated or not at all due to the absorption and scattering of the above cells and EPS matrix. In this case, the reducing effect became weaker with increasing irradiation dose, an effect that could be described by a Weibull model or an exponential decay with tail (“log-lin + tail”).

To judge which of these mathematical approaches best fits the experimental results, the RMSE (residual mean squared errors) was determined by the tool Bioinactivation for mono-species biofilms of *P. aeruginosa*, *E. coli*, *A. baumannii*., *S. Typhimurium*, *S. aureus*, *L. monocytogenes*, and *C. albicans* and all Gram-positive and Gram-negative bacteria, fungi, and multi-species biofilms. The results for the UVC region can be found in Table 5 and for violet light in Table 6. In most cases the highly scattered biofilm reduction results were best described by a Weibull model but the simple exponential (“log-lin”) was often not much worse. Therefore, the fitted curves of both models were added to Figure 1, Figure 2, Figure 3 and Figure 4. In both cases large deviations between model and experimental data could be observed.

Additionally, MANOVA tests were applied to analyze whether the data (scatter plots) for the different groups were significantly different (α = 0.05). For both wavelength ranges mono-species Gram+ bacterial biofilm reduction was compared separately to mono-species Gram- bacterial and fungal biofilm reductions and the reduction in the multi-species biofilms. Additionally, Gram- bacterial and fungal mono-species biofilm reductions were compared to each other and to the multi-species biofilm results. Prior to the MANOVA calculations, the different data sets were checked for normality by the Kolmogorov–Smirnov tests (α = 0.05). Normality was excluded for 4 of the 24 different data sets. However, as Wilks’ lambda (WL) is robust to violation of normality [18,19,20,21] and was here complemented by other MANOVA tests, the tests were performed and analyzed.

The result of all single comparisons was similar: The differences between these groups were significant but only small or even not large enough to be statistically significant at all (α = 0.05). Details are presented in Table 7 and Table 8.

Additionally, the experimental data was divided by substrate material into three groups: metal (steel, titanium), plastics (PS, PE, PP, PMMA, PVC) and other substrate materials. Further MANOVA tests were applied to judge whether the results from metal and plastic substrate revealed a significant difference for UVC or violet biofilm irradiation. For the UVC irradiation the difference was not significant (WL: ns, PT: ns, HL: ns, RM: ns). For the violet region (400–420 nm) the MANOVA test also resulted in a non-significant difference between the metal and plastic substrate groups (WL: ns, PT: ns, HL: ns, RM: ns).

## 4. Discussion

The observed differences in radiation sensitivity are very large. For example, the log-reductions achieved for *P. aeruginosa* biofilms with violet light in the dose range of 200–300 J/cm^2^ vary by more than 5 orders of magnitude. This scattering of results makes it difficult to reach concrete statements on biofilm sensitivity or differences in the sensitivity of different microorganisms or on the influence of the biofilm substrate material.

Part of this scatter could have been caused by the irradiation setup. More than 25 investigations for biofilm culturing and irradiation were performed in 96-well MTPs. These MTPs are well suited for biofilm cultivation, but poorly qualified for biofilm irradiation, as the relatively high walls provide unavoidable shading. Even an unirradiated area of only 1% of the total surface makes evaluations of log-reductions in the range of 2 or higher mostly meaningless. In addition, the varying degrees of shading cause further scattering of the irradiation results. Angarano et al. [127] were the only authors that mentioned MTP well shadowing.

Some of the studies applied irradiation intensities above or even far above 100 mW/cm^2^, which is rather high and might lead to heating especially for biofilm samples on plastics that are not good heat conductors. For comparison, at noon in summer, the total solar irradiation is also at about 100 mW/cm^2^ and absorbing materials get very hot. Prasad and Roopesh irradiated biofilms with 290 mW/cm^2^, and though their substrate was steel, which probably worked as a good heat conductor and spreader, they observed temperatures above 50 °C [123]. Other authors applied even higher intensities with biofilms on substrates that exhibited no good heat conducting properties; therefore, the lethal mechanism might be due to heat rather than photoinactivation. This might not only happen at intensities above 100 mW/cm^2^, especially for biofilms on plastic substrates like MTPs. Therefore, the biofilm temperature should be checked.

Gora et al. [11] have addressed some other important issues that may be the reason for the observed differences. These include biofilm age and biofilm cultivation conditions. In studies included in this review, the ages of most mature biofilms were between 24 and 72 h. The reported biofilm cell densities and thicknesses varied between 10^5^ and 10^9^ cells/cm^2^ and 0.43 (probably a mono-layer biofilm) and 200 µm, respectively, which reveal differences in several orders of magnitude between seemingly similar biofilms because deeper biofilm layers might be radiation-protected by the absorption and scattering of the upper layers.

In addition, Gora et al. drew attention to a possible VBNC (viable but not culturable) problem, in other words, even in irradiated biofilms there could be cells that are not dead but cannot be propagated when detected on agar plates, for example. They also issued a warning about the influence of various techniques for determining biofilm reduction, such as plating or crystal violet staining [11].

For practical reasons, we ignored these problems in the way that we included all data obtained with all MTPs and other substrates and all irradiation intensities and hoped that the various effects would partially compensate for each other. The possible influence of shadow formation on biofilm reduction may also be lost in the large scattering of results due to different biofilm growth conditions, different irradiation parameters, and different surfaces, both in terms of reflective properties and roughness of the surface materials.

Further scattering might be caused by the combination of data from different strains of microorganisms. It seems that biofilm reduction is possible with all wavelengths between 200 and 525 nm, if the irradiance is high enough. The best model to describe the relation between dose and log-reduction seems to be the Weibull model but the simple exponential approximation is not much worse.

Since the various irradiation tests were carried out on different materials, these biofilm substrates could also have an influence on the biofilm sensitivity, but this has not yet been apparent in the highly scattered individual results.

Pousty et al. [117] stated that the higher log-reduction doses were feasible with “blue” (violet) light than with UVC radiation, but in Figure 3 maximum log-reduction of about seven log-levels were observed for both ranges. However, the required total doses were much higher for the violet/blue spectral range. If the average log-reduction doses for Gram+ bacteria, Gram- bacteria and fungi according to the given trend lines for UVC (950 mJ/cm^2^, 770 mJ/cm^2^ and 1439 mJ/cm^2^) and violet light (769 J/cm^2^, 85.5 J/cm^2^ and 107.5 J/cm^2^) are compared, it becomes clear that UVC is much more efficient by two to three orders of magnitude.

We might add that Figure 3 leads to the impression that violet light (400–420 nm) has a stronger impact than blue light (440–480 nm), as only about twenty percent of the blue light irradiation report inactivations of more than 2 log-levels.

Therefore, there is no clear best biofilm irradiation wavelength or even spectral region, even if efficiency is ignored. According to Ma et al. it is UVC [87], for Argyraki et al. [100] it is UVB, and for Pousty et al. it is visible blue (violet) light [117].

Besides a few claims of an irradiation threshold that inhibits biofilm formation, it seems more likely that there is no UVC or visible light surface irradiance that totally prevents biofilm formation—or at least it has not been found, yet.

Surprisingly, there are less than 10 investigations with UVB or UVA radiation, though Argyraki et al. reached a better biofilm reduction with UVB than UVC [100]. Also unexpectedly, there are only a few UVC irradiations of many important (mono-species) biofilms with pathogens like some dreaded ESKAPE bacteria (ESKAPE: *Enterococcus faecium*, *Staphylococcus aureus*, *Klebsiella pneumoniae*, *Acinetobacter baumannii*, *Pseudomonas aeruginosa* and *Enterobacter* spp.).

## 5. Conclusions

In general, radiation in the 200–525 nm range (UVC—blue/green light) appears to be able to slow down biofilm growth or even reduce biofilms if the irradiation is strong enough.

The questions we raised in the introduction are answered as follows:

- The irradiation of water reduces or delays biofilm formation only in some situations or for some water conditions.
- Irradiation of surfaces reduces or delays biofilm formation. This is true for the spectral range 200–525 nm if the irradiation intensity is high enough.
- UVC seems to be much more efficient in biofilm reduction than visible blue/violet light, but it seems still unclear which wavelength is best for biofilm irradiation and reduction.
- Multi-species biofilms might be more irradiation resistant than mono-species biofilms, but the difference seems to be small.
- Compared to the scattering of the results, there are no large differences between the photosensitivities of Gram+ bacterial, Gram- bacterial, and fungal biofilms.
- Cells in biofilms are more radiation resistant than planktonic cells.
- The impact of the biofilm substrate seems to be rather low.

Much research seems to be still “missing”—even UVC experiments on *S. aureus* and other ESKAPE pathogens are quite rare, but also biofilm irradiations in the UVB and UVA region. Especially 96-well MTPs should be avoided for future biofilm irradiation research—at least if the biofilm is irradiated within a well. Otherwise, we support the best practice recommendations for future biofilm irradiation experiments of Gora et al. [11].

## Figures and Tables

**Table 1:** List of the microorganisms used in the different biofilm inactivation studies and targeted in this meta-analysis. The abbreviated organism names are used in the body text, figures, and tables hereafter. Gram-positive (GP) and Gram-negative (GN) indicates response to Gram staining; EFS displays the organisms’ capacity to form endospores.

Microorganism	Abbreviation	Taxonomic Class	Comments
**Bacteria**			
*Actinomyces naeslundii*	*A. naeslundii*	Actinomycetes	GP, anaerobe or microaerophilic
*Aeromonas australiensis*	*A. australiensis*	Gammaproteobacteria	GN, anaerobe
*Acinetobacter baumannii*	*A. baumannii*	Gammaproteobacteria	GN,
*Aeromonas hydrophilia*	*A. hydrophilia*	Gammaproteobacteria	GN, facultative anaerobe
*Aggregatibacter actinomycetemcomitans*	*A. actinomycetem-comitans*	Gammaproteobacteria	GN, facultative anaerobe
*Alicyclobacillus acidocaldarius*	*A. acidocaldarius*	Bacilli	GP, strict aerobic, ESF
*Alicyclobacillus acidoterrestris*	*A. acidoterrestris*	Bacilli	GP, strict aerobic, ESF
*Alicyclobacillus cycloheptanicus*	*A. cycloheptanicus*	Bacilli	GP, strict aerobic, ESF
*Alicyclobacillus herbarius*	*A. herbarius*	Bacilli	GP, strict aerobic, ESF
*Bacillus cereus*	*B. cereus*	Bacilli	GP, aerobic or facultative anaerobe, ESF
*Bacillus thuringiensis*	*B. thuringiensis*	Bacilli	GP, aerobic, ESF
*Burkholderia multivorans*	*B. multivorans*	Betaproteobacteria	GN, aerobic,
*Cupriavidus metallidurans*	*C. metallidurans*	Betaproteobacteria	GN, aerobic
*Enterococcus faecalis*	*E. faecalis*	Bacilli	GP, facultative anaerobe
*Escherichia coli*	*E. coli*	Gammaproteobacteria	GN, facultative anaerobe
*Flavobacterium breve*	*F. breve*	Flavobacteriia	GN, strict aerobic
*Fusobacterium nucleatum*	*F. nucleatum*	Fusobacteriia	GN, anaerobe
*Klebsiella oxytoca*	*K. oxytoca*	Gammaproteobacteria	GN, facultative anaerobe
*Klebsiella pneumoniae*	*K. pneumoniae*	Gammaproteobacteria	GN, facultative anaerobe
*Lactobacillus brevis*	*L. brevis*	Bacilli	GP, facultative anaerobe
*Leuconostoc citreum*	*L. citreum*	Bacilli	GP, facultative anaerobe
*Listeria monocytogenes*	*L. monocytogenes*	Bacilli	GP, facultative anaerobe
*Methylobacterium fujisawaense*	*M.* *fujisawaense*	Alphaproteobacteria	GN, facultative anaerobe
*Moraxella catarrhalis*	*M. catarrhalis*	Gammaproteobacteria	GN, aerobic
*Pediococcus acidilacti*	*P. acidilacti*	Bacilli	GP, facultative anaerobe
*Porphyromonas gingivalis*	*P. gingivalis*	Bacterioidia	GN, anaerobe
*Propionibacterium acnes* ^1^	*P. acnes*	Actinomycetes	GP, aerotolerant,
*Proteus mirabilis*	*P. mirabilis*	Gammaproteobacteria	GN, facultative anaerobe
*Pseudomonas aeruginosa*	*P. aeruginosa*	Gammaproteobacteria	GN, facultative anaerobe
*Pseudomonas fluorescens*	*P. fluorescens*	Gammaproteobacteria	GN, facultative anaerobe
*Ralstonia insidiosa*	*R. insidiosa*	Betaproteobacteria	GN, aerobic
*Salmonella* Typhimurium	*S.* Typhimurium	Gammaproteobacteria	GN, facultative anaerobe
*Staphylococcus aureus*	*S. aureus*	Bacilli	GP, facultative anaerobe
*Staphylococcus epidermis*	*S. epidermis*	Bacilli	GP, facultative anaerobe
*Streptococcus mutans*	*S. mutans*	Bacilli	GP, facultative anaerobe
*Streptococcus sanguinis*	*S. sanguinis*	Bacilli	GP, facultative anaerobe
*Vibrio parahaemolyticus*	*V. parahaemo-lyticus*	Gammaproteobacteria	GN, facultative anaerobe
*Vibrio vulnificus*	*V. vulnificus*	Gammaproteobacteria	GN, facultative anaerobe
**Fungi**			
*Aspergillus niger*	*A. niger*	Eurotiomycetes	Aerobic
*Brettanomyces bruxellensis*	*B. bruxellensis*	Pichiomycetes	Facultative anaerobe
*Candida albicans*	*C. albicans*	Pichiomycetes	Facultative anaerobe
*Candida auris* ^2^	*C. auris*	Pichiomycetes	Facultative anaerobe
*Candida glabrata* ^3^	*C. glabrata*	Saccharomycetales	Facultative anaerobe
*Candida parapsilosis*	*C. parapsilosis*	Pichiomycetes	Anaerobe
*Cryptococcus neoformans*	*C. neoformans*	Tremellomycetes	Obligate aerobic
*Fusarium solani*	*F. solani*	Sordariomycetes	Facultative anaerobe
*Penicillium glaucum*	*P. glaucum*	Eurotiomycetes	Facultative anaerobe
**Algae**			
*Navicula incerta*	*N. incerta*	Bacillariophyceae	

^1^ new name: Cutibacterium acnes, ^2^ new name: Candidozyma auris, ^3^ new name: Nakaseomyces glabratus.

**Table 5:** RMSE (residual mean squared errors) for the collected UVC data for the different microorganisms and groups of microorganisms calculated with the Bioinactivation tool [17].

RMSE for:	“Log-Lin” Bigelow 1920 [13]	“Log-Lin + Tail” Geeraerd 2005 [15]	“Weibull” Mafart 2002 [14]
*Pseudomonas aeruginosa*	1.647	1.09	1.02
*Escherichia coli*	2.121	1.544	1.533
*Staphylococcus aureus*	2.078	0.895	0.94
*Salmonella* Typhimurium	2.592	1.59	1.57
*Listeria monocytogenes*	0.9854	1.00	0.9433
Gram+ bacteria	1.651	1.104	1.115
Gram- bacteria	1.986	1.727	1.261
fungi	2.053	1.414	0.911
multi-species biofilm	2.479	1.319	1.41

**Table 6:** RMSE (residual mean squared errors) for the collected violet data for the different microorganisms and groups of microorganisms calculated with the Bioinactivation tool [17].

RMSE for:	“Log-Lin” Bigelow 1920 [13]	“Log-Lin + Tail” Geeraerd 2005 [15]	“Weibull” Mafart 2002 [14]
*P. aeruginosa*	1.294	1.326	1.293
*E. coli*	1.033	1.27	1.076
*A. baumannii*	1.266	1.82	1.267
*S. aureus*	0.8005	0.8185	0.8003
*L. monocytogenes*	1.093	0.84	0.6989
*C. albicans*	0.7538	0.97	0.7496
Gram+ bacteria	1.578	0.907	0.9316
Gram- bacteria	1.521	1.308	1.398
fungi	1.546	1.341	1.335
multi-species biofilm	0.1343	0.155	0.1404

**Table 7:** Comparing mono-species Gram+, Gram- bacterial and fungal and multi-species biofilm UVC irradiation results for significant differences by Wilks’s lambda MANOVA tests. (α = 0.05, ns: not significant, ssd: significant small difference, PT: Pillai’s trace, HL: Hotteling–Lawley trace, RM: Roy’s maximum root).

	Gram+ Bacteria	Gram- Bacteria	Fungi	Multi-Species
**Gram+ bacteria**		**ns** PT: ns, HL: ns, RM: ns	**ssd** PT: ns, HL: ssd, RM: ssd	**ssd** PT: ns, HL: ssd, RM: ssd
**Gram- bacteria**	**ns** PT: ns, HL: ns, RM: ns		**ns** PT: ns, HL: ns, RM: ns	**ns** PT: ns, HL: ns, RM: ns
**fungi**	**ssd** PT: ns, HL: ssd, RM: ssd	**ns** PT: ns, HL: ns, RM: ns		**ns** PT: ns, HL: ns, RM: ns
**multi-species**	**ssd** PT: ns, HL: ssd, RM: ssd	**ns** PT: ns, HL: ns, RM: ns	**ns** PT: ns, HL: ns, RM: ns	

**Table 8:** Comparing mono-species Gram+, Gram- bacterial and fungal and multi-species biofilm visible violet irradiation results for significant differences by MANOVA tests. (α = 0.05, ns: not significant, ssd: significant small difference, PT: Pillai’s trace, HL: Hotteling–Lawley trace, RM: Roy’s maximum root).

	Gram+ Bacteria	Gram- Bacteria	Fungi	Multi-Species
**Gram+ bacteria**		**ssd** PT: ns, HL: ssd, RM: ssd	**ns** PT: ns, HL: ns, RM: ns	**ns** PT: ns, HL: ns, RM: ns
**Gram- bacteria**	**ssd** PT: ns, HL: ssd, RM: ssd		**ssd** PT: ssd, HL: ssd, RM: ssd	**ns** PT: ns, HL: ns, RM: ns
**fungi**	**ns** PT: ns, HL: ns, RM: ns	**ssd** PT: ssd, HL: ssd, RM: ssd		**ns** PT: ns, HL: ns, RM: ns
**multi-species**	**ns** PT: ns, HL: ns, RM: ns	**ns** PT: ns, HL: ns, RM: ns	**ns** PT: ns, HL: ns, RM: ns	

## Data Availability

No new data were created or analyzed in this study. Data sharing is not applicable to this article.

## References

[1] Funari R., Shen A.Q. (2022). Detection and Characterization of Bacterial Biofilms and Biofilm-Based Sensors. ACS Sens..

[2] Hall-Stoodley L., Costerton J.W., Stoodley P. (2004). Bacterial biofilms: From the natural environment to infectious diseases. Nat. Rev. Microbiol..

[3] Flemming H.-C., Wingender J., Szewzyk U., Steinberg P., Rice S.A., Kjelleberg S. (2016). Biofilms: An emergent form of bacterial life. Nat. Rev. Microbiol..

[4] Cámara M., Green W., MacPhee C.E., Rakowska P.D., Raval R., Richardson M.C., Slater-Jefferies J., Steventon K., Webb J.S. (2022). Economic significance of biofilms: A multidisciplinary and cross-sectoral challenge. NPJ Biofilms Microbiomes.

[5] Li Y., Narayanan M., Shi X., Chen X., Li Z., Ma Y. (2024). Biofilms formation in plant growth-promoting bacteria for alleviating agro-environmental stress. Sci. Total Environ..

[6] Flemming H.-C., van Hullebusch E.D., Neu T.R., Nielsen P.H., Seviour T., Stoodley P., Wingender J., Wuertz S. (2023). The biofilm matrix: Multitasking in a shared space. Nat. Rev. Microbiol..

[7] Jagger J. (1968). Introduction to Research in Ultraviolet Photobiology. Photochem. Photobiol..

[8] de Jager T.L., Cockrell A.E., Du Plessis S.S. (2017). Ultraviolet Light Induced Generation of Reactive Oxygen Species. Adv. Exp. Med. Biol..

[9] Tomb R.M., White T.A., Coia J.E., Anderson J.G., MacGregor S.J., Maclean M. (2018). Review of the Comparative Susceptibility of Microbial Species to Photoinactivation Using 380-480 nm Violet-Blue Light. Photochem. Photobiol..

[10] Hessling M., Spellerberg B., Hoenes K. (2016). Photoinactivation of bacteria by endogenous photosensitizers and exposure to visible light of different wavelengths—A review on existing data. FEMS Microbiol. Lett..

[11] Gora S.L., Ma B., Lanzarini-Lopes M., Torkzadeh H., Zhao Z., Ley Matthews C., Westerhoff P., Linden K., Barbeau B., Simons R. (2024). Control of biofilms with UV light: A critical review of methodologies, research gaps, and future directions. Environ. Sci. Water Res. Technol..

[12] Grzelak A., Rychlik B., Bartosz G. (2001). Light-dependent generation of reactive oxygen species in cell culture media. Free. Radic. Biol. Med..

[13] Bigelow W.D. (1921). The logarithmic nature of thermal death time curves. J. Infect. Dis..

[14] Mafart P., Couvert O., Gaillard S., Leguerinel I. (2002). On calculating sterility in thermal preservation methods: Application of the Weibull frequency distribution model. Int. J. Food Microbiol..

[15] Geeraerd A.H., Valdramidis V.P., van Impe J.F. (2005). GInaFiT, a freeware tool to assess non-log-linear microbial survivor curves. Int. J. Food Microbiol..

[16] Garre A., Fernández P.S., Lindqvist R., Egea J.A. (2017). Bioinactivation: Software for modelling dynamic microbial inactivation. Food Res. Int..

[17] Garre A., Clemente-Carazo M., Fernández P.S., Lindqvist R., Egea J.A. (2018). Bioinactivation FE: A free web application for modelling isothermal and dynamic microbial inactivation. Food Res. Int..

[18] O’Brien P.N., Parenté F.J., Schmitt C.J. (1982). A monte carlo study on the robustness of four MANOVA criterion tests. J. Stat. Comput. Simul..

[19] Haase R.F., Ellis M.V. (1987). Multivariate analysis of variance. J. Couns. Psychol..

[20] Rencher A.C. (2005). A Review of “Methods of Multivariate Analysis, Second Edition”. IIE Trans..

[21] Ateş C., Kaymaz Ö., Kale H.E., Tekindal M.A. (2019). Comparison of Test Statistics of Nonnormal and Unbalanced Samples for Multivariate Analysis of Variance in terms of Type-I Error Rates. Comput. Math. Methods Med..

[22] Statistics Kingdom Statistics Online. https://www.statskingdom.com/index.html.

[23] Momba M.N.B., Cloete T.E., Venter S.N., Kfir R. (1998). Evaluation of the impact of disinfection processes on the formation of biofilms in potable surface water distribution systems. Water Sci. Technol..

[24] Schwartz T., Hoffmann S., Obst U. (2003). Formation of natural biofilms during chlorine dioxide and u.v. disinfection in a public drinking water distribution system. J. Appl. Microbiol..

[25] Marconnet C., Houari A., Seyer D., Djafer M., Coriton G., Heim V., Di Martino P. (2011). Membrane biofouling control by UV irradiation. Desalination.

[26] Wenjun S., Wenjun L. (2009). Impact of the Ultraviolet Disinfection Process on Biofilm Control in a Model Drinking Water Distribution System. Environ. Eng. Sci..

[27] Wu Y.-H., Chen Z., Li X., Wang Y.-H., Liu B., Chen G.-Q., Luo L.-W., Wang H.-B., Tong X., Bai Y. (2021). Effect of ultraviolet disinfection on the fouling of reverse osmosis membranes for municipal wastewater reclamation. Water Res..

[28] Pozos N., Scow K., Wuertz S., Darby J. (2004). UV disinfection in a model distribution system: Biofilm growth and microbial community. Water Res..

[29] Saidi N., Kouki S., Mehri I., Ben Rejeb A., Belila A., Hassen A., Ouzari H. (2011). Biofilm and siderophore effects on secondary waste water disinfection. Curr. Microbiol..

[30] Vankerckhoven E., Verbessem B., Crauwels S., Declerck P., Muylaert K., Willems K.A., Rediers H. (2011). Exploring the potential synergistic effects of chemical disinfectants and UV on the inactivation of free-living bacteria and treatment of biofilms in a pilot-scale system. Water Sci. Technol..

[31] Rand J.L., Sharafimasooleh M., Walsh M.E. (2013). Effect of water hardness and pipe material on enhanced disinfection with UV light and chlorine. J. Water Supply Res. Technol..

[32] Jungfer C., Friedrich F., Varela Villarreal J., Brändle K., Gross H.-J., Obst U., Schwartz T. (2013). Drinking water biofilms on copper and stainless steel exhibit specific molecular responses towards different disinfection regimes at waterworks. Biofouling.

[33] Yu W., Campos L.C., Graham N. (2016). Application of pulsed UV-irradiation and pre-coagulation to control ultrafiltration membrane fouling in the treatment of micro-polluted surface water. Water Res..

[34] Benito A., Garcia G., Gonzalez-Olmos R. (2017). Fouling reduction by UV-based pretreatment in hollow fiber ultrafiltration membranes for urban wastewater reuse. J. Membr. Sci..

[35] Metz D.H., Reynolds K., Meyer M., Dionysiou D.D. (2011). The effect of UV/H_2_O_2_ treatment on biofilm formation potential. Water Res..

[36] Harif T., Elifantz H., Margalit E., Herzberg M., Lichi T., Minz D. (2011). The effect of UV pre-treatment on biofouling of BWRO membranes: A field study. Desalination Water Treat..

[37] Kviatkovski I., Mamane H., Lakretz A., Sherman I., Beno-Moualem D., Minz D. (2018). Resistance of a multiple-isolate marine culture to ultraviolet C irradiation: Inactivation vs biofilm formation. Lett. Appl. Microbiol..

[38] Lakretz A., Ron E.Z., Mamane H. (2010). Biofouling control in water by various UVC wavelengths and doses. Biofouling.

[39] Lakretz A., Ron E.Z., Mamane H. (2011). Biofilm control in water by a UV-based advanced oxidation process. Biofouling.

[40] Friedman L., Harif T., Herzberg M., Mamane H. (2016). Mitigation of Biofilm Colonization on Various Surfaces in a Model Water Flow System by Use of UV Treatment. Water Air Soil Pollut.

[41] Lakretz A., Mamane H., Asa E., Harif T., Herzberg M. (2018). Biofouling control by UV/H_2_O_2_ pretreatment for brackish water reverse osmosis process. Environ. Sci. Water Res. Technol..

[42] Lund V., Ormerod K. (1995). The influence of disinfection processes on biofilm formation in water distribution systems. Water Res..

[43] Otaki M., Takizawa S., Ohgaki S. (1998). Control and modeling of membrane fouling due to microorganism growth by UV pretreatment. Water Sci. Technol..

[44] Sperle P., Khan M.S., Skibinski B., Wurzbacher C., Drewes J.E. (2023). Optimizing UVC-disinfection using LEDs as an energy efficient pre-treatment for biofouling control in spiral-wound membrane systems. Desalination.

[45] Sperle P., Wurzbacher C., Drewes J.E., Skibinski B. (2020). Reducing the Impacts of Biofouling in RO Membrane Systems through In Situ Low Fluence Irradiation Employing UVC-LEDs. Membranes.

[46] Randall T., Shlomo I., Wells E., Real B., Ma B., Linden Y., Gamboa J., Friedler E., Linden K.G. (2024). Evaluation of UVLED disinfection for biofouling control during distribution of wastewater effluent. Water Reuse.

[47] Karim N.S., Sarker N.R., Asker D., Hatton B., Bilton A.M. (2025). Can UVC-LEDs mitigate biofouling in community-scale photovoltaic-powered reverse osmosis systems?. Water Supply.

[48] Zhao Z., Rho H., Shapiro N., Ling L., Perreault F., Rittmann B., Westerhoff P. (2023). Biofilm inhibition on surfaces by ultraviolet light side-emitted from optical fibres. Nat. Water.

[49] Zhao Z., Luo Y.-H., Wang T.-H., Sinha S., Ling L., Rittmann B., Alvarez P., Perreault F., Westerhoff P. (2023). Phenotypic and Transcriptional Responses of Pseudomonas aeruginosa Biofilms to UV-C Irradiation via Side-Emitting Optical Fibers: Implications for Biofouling Control. Environ. Sci. Technol..

[50] Hunsucker K.Z., Braga C., Gardner H., Jongerius M., Hietbrink R., Salters B., Swain G. (2019). Using ultraviolet light for improved antifouling performance on ship hull coatings. Biofouling.

[51] Ryan E., Turkmen S., Benson S. (2020). An Investigation into the application and practical use of (UV) ultraviolet light technology for marine antifouling. Ocean Eng..

[52] Piola R., Salters B., Grandison C., Ciacic M., Hietbrink R. (2016). Assessing the Use of Low Voltage UV-Light Emitting Miniature LEDs for Marine Biofouling Control: Technical Report.

[53] Salters B., Piola R. (2017). UVC Light for Antifouling. Mar. Technol. Soc. J..

[54] Lanzarini-Lopes M., Zhao Z., Perreault F., Garcia-Segura S., Westerhoff P. (2020). Germicidal glowsticks: Side-emitting optical fibers inhibit Pseudomonas aeruginosa and Escherichia coli on surfaces. Water Res..

[55] Chen H., Moraru C.I. (2023). Exposure to 222 nm far UV-C effectively inactivates planktonic foodborne pathogens and inhibits biofilm formation. Innov. Food Sci. Emerg. Technol..

[56] Patil J.S., Kimoto H., Kimoto T., Saino T. (2007). Ultraviolet radiation (UV-C): A potential tool for the control of biofouling on marine optical instruments. Biofouling.

[57] Mariita R.M., Davis J.H., Lottridge M.M., Randive R.V. (2022). Shining light on multi-drug resistant *Candida auris*: Ultraviolet-C disinfection, wavelength sensitivity, and prevention of biofilm formation of an emerging yeast pathogen. Microbiologyopen.

[58] Torkzadeh H., Zodrow K.R., Bridges W.C., Cates E.L. (2021). Quantification and modeling of the response of surface biofilm growth to continuous low intensity UVC irradiation. Water Res..

[59] Torkzadeh H., Cates E.L. (2021). Biofilm growth under continuous UVC irradiation: Quantitative effects of growth conditions and growth time on intensity response parameters. Water Res..

[60] Vollmerhausen T.L., Conneely A., Bennett C., Wagner V.E., Victor J.C., O’Byrne C.P. (2017). Visible and UVA light as a potential means of preventing *Escherichia coli* biofilm formation in urine and on materials used in urethral catheters. J. Photochem. Photobiol. B.

[61] European Union (2006). Directive 2006/25/EC of the European Parliament and of the Council on the minimum health and safety requirements regarding the exposure of workers to risks arising from physical agents (artificial optical radiation). Off. J. Eur. Union.

[62] Butement J.T., Noel D.J., Bryant C.A., Wilks S.A., Eason R.W. (2022). A light-guiding urinary catheter for the inhibition of Proteus mirabilis biofilm formation. Front. Microbiol..

[63] Braga C., Hunsucker K., Erdogan C., Gardner H., Swain G. (2020). The Use of a UVC Lamp Incorporated with an ROV to Prevent Biofouling: A Proof-of-Concept Study. Mar. Technol. Soc. J..

[64] Braga C., Hunsucker K., Gardner H., Swain G. (2020). A novel design to investigate the impacts of UV exposure on marine biofouling. Appl. Ocean. Res..

[65] Disalvo L.H., Cobet A.B. (1974). Control of an estuarine microfouling sequence on optical surfaces using low-intensity ultraviolet irradiation. Appl. Microbiol..

[66] Bak J., Ladefoged S.D., Begovic T., Winding A. (2010). UVC fluencies for preventative treatment of Pseudomonas aeruginosa contaminated polymer tubes. Biofouling.

[67] Hoeher P.A., Zenk O., Cisewski B., Boos K., Groeger J. (2023). UVC-Based Biofouling Suppression for Long-Term Deployment of Underwater Cameras. IEEE J. Ocean. Eng..

[68] Bueley C., Olender D., Bocking B. (2014). In-Situ Trial of Uv-C as an Antifoulant to Reduce Biofouling Induced Measurement Error. J. Ocean. Technol..

[69] Alidokht L., Fitzpatrick K., Butler C., Hunsucker K.Z., Braga C., Maza W.A., Fears K.P., Arekhi M., Lanzarini-Lopes M. (2024). UV emitting glass: A promising strategy for biofilm inhibition on transparent surfaces. Biofilm.

[70] Whitworth P., Aldred N., Reynolds K.J., Plummer J., Duke P.W., Clare A.S. (2022). Importance of Duration, Duty-Cycling and Thresholds for the Implementation of Ultraviolet C in Marine Biofouling Control. Front. Mar. Sci..

[71] MacKenzie A.F., Maltby E.A., Harper N., Bueley C., Olender D., Wyeth R.C. (2019). Periodic ultraviolet-C illumination for marine sensor antifouling. Biofouling.

[72] Purvis K., Curnew K.H., Trevors A.L., Hunter A.T., Wilson E.R., Wyeth R.C. (2022). Single Ultraviolet-C light treatment of early stage marine biofouling delays subsequent community development. Biofouling.

[73] Gomez G.F., Huang R., MacPherson M., Ferreira Zandona A.G., Gregory R.L. (2016). Photo Inactivation of Streptococcus mutans Biofilm by Violet-Blue light. Curr. Microbiol..

[74] Li X., Kim M.-J., Bang W.-S., Yuk H.-G. (2018). Anti-biofilm effect of 405-nm LEDs against Listeria monocytogenes in simulated ready-to-eat fresh salmon storage conditions. Food Control.

[75] Martegani E., Bolognese F., Trivellin N., Orlandi V.T. (2020). Effect of blue light at 410 and 455 nm on Pseudomonas aeruginosa biofilm. J. Photochem. Photobiol. B.

[76] Rupel K., Zupin L., Ottaviani G., Bertani I., Martinelli V., Porrelli D., Vodret S., Vuerich R., Passos da Silva D., Bussani R. (2019). Blue laser light inhibits biofilm formation in vitro and in vivo by inducing oxidative stress. NPJ Biofilms Microbiomes.

[77] Bumah V.V., Masson-Meyers D.S., Enwemeka C.S. (2020). Pulsed 450 nm blue light suppresses MRSA and Propionibacterium acnes in planktonic cultures and bacterial biofilms. J. Photochem. Photobiol. B.

[78] Bapat P., Singh G., Nobile C.J. (2021). Visible Lights Combined with Photosensitizing Compounds Are Effective against Candida albicans Biofilms. Microorganisms.

[79] Sun W., Shi S., Chen J., Zhao W., Chen T., Li G., Zhang K., Yu B., Liu D., Chen Y. (2022). Blue Light Signaling Regulates *Escherichia coli* W1688 Biofilm Formation and l-Threonine Production. Microbiol. Spectr..

[80] Halstead F.D., Thwaite J.E., Burt R., Laws T.R., Raguse M., Moeller R., Webber M.A., Oppenheim B.A. (2016). Antibacterial Activity of Blue Light against Nosocomial Wound Pathogens Growing Planktonically and as Mature Biofilms. Appl. Environ. Microbiol..

[81] Song H.-H., Lee J.-K., Um H.-S., Chang B.-S., Lee S.-Y., Lee M.-K. (2013). Phototoxic effect of blue light on the planktonic and biofilm state of anaerobic periodontal pathogens. J. Periodontal. Implant Sci..

[82] Martinez L.R., Casadevall A. (2007). Cryptococcus neoformans biofilm formation depends on surface support and carbon source and reduces fungal cell susceptibility to heat, cold, and UV light. Appl. Environ. Microbiol..

[83] Prado D.B.d., Szczerepa M.M.D.A., Capeloto O.A., Astrath N.G.C., Santos N.C.A.D., Previdelli I.T.S., Nakamura C.V., Mikcha J.M.G., de Abreu Filho B.A. (2019). Effect of ultraviolet (UV-C) radiation on spores and biofilms of *Alicyclobacillus* spp. in industrialized orange juice. Int. J. Food Microbiol..

[84] Liu Z., Hu S., Soteyome T., Bai C., Liu J., Wang Z., Kjellerup B.V., Xu Z. (2021). Intense pulsed light for inactivation of foodborne gram-positive bacteria in planktonic cultures and bacterial biofilms. LWT-Food Sci. Technol..

[85] Li X., Gu N., Ye Y., Lan H., Peng F., Peng G. (2022). Intense pulsed light for inactivating planktonic and biofilm molds in food. Front. Microbiol..

[86] Shi Y.-G., Jiang L., Lin S., Jin W.-G., Gu Q., Chen Y.-W., Zhang K., Ettelaie R. (2022). Ultra-efficient antimicrobial photodynamic inactivation system based on blue light and octyl gallate for ablation of planktonic bacteria and biofilms of *Pseudomonas fluorescens*. Food Chem..

[87] Ma B., Seyedi S., Wells E., McCarthy D., Crosbie N., Linden K.G. (2022). Inactivation of biofilm-bound bacterial cells using irradiation across UVC wavelengths. Water Res..

[88] Angarano V., Akkermans S., Smet C., Chieffi A., van Impe J.F.M. (2020). The potential of violet, blue, green and red light for the inactivation of *P. fluorescens* as planktonic cells, individual cells on a surface and biofilms. Food Bioprod. Process..

[89] Jones C.C., Valdeig S., Sova R.M., Weiss C.R. (2016). Inside-out Ultraviolet-C Sterilization of Pseudomonas aeruginosa Biofilm In Vitro. Photochem. Photobiol..

[90] Liu X., Chang Q., Ferrer-Espada R., Leanse L.G., Goh X.S., Wang X., Gelfand J.A., Dai T. (2020). Photoinactivation of Moraxella catarrhalis Using 405-nm Blue Light: Implications for the Treatment of Otitis Media. Photochem. Photobiol..

[91] Córdova-Alcántara I.M., Venegas-Cortés D.L., Martínez-Rivera M.Á., Pérez N.O., Rodriguez-Tovar A.V. (2019). Biofilm characterization of *Fusarium solani* keratitis isolate: Increased resistance to antifungals and UV light. J. Microbiol..

[92] Pezzoni M., Pizarro R.A., Costa C.S. (2014). Protective role of extracellular catalase (KatA) against UVA radiation in *Pseudomonas aeruginosa* biofilms. J. Photochem. Photobiol. B.

[93] Shen L., Chen W., He J., Luo X., Mei Y., Zhang B. (2024). Effective management of pre-existing biofilms using UV-LED through inactivation, disintegration and peeling. J. Hazard. Mater..

[94] Palma F., Díaz-Navarro M., Visedo A., Sanz-Ruíz P., Brandi G., Schiavano G.F., Guembe M. (2025). Assessment of the anti-biofilm effect of UV-C irradiation (254 nm) against healthcare associated infections related microorganisms. Front. Microbiol..

[95] Plattfaut I., Demir E., Fuchs P.C., Schiefer J.L., Stürmer E.K., Brüning A.K.E., Opländer C. (2021). Characterization of Blue Light Treatment for Infected Wounds: Antibacterial Efficacy of 420, 455, and 480 nm Light-Emitting Diode Arrays Against Common Skin Pathogens Versus Blue Light-Induced Skin Cell Toxicity. Photobiomodulation Photomed. Laser Surg..

[96] Yang Y., Ma S., Xie Y., Wang M., Cai T., Li J., Guo D., Zhao L., Xu Y., Liang S. (2020). Inactivation of Pseudomonas aeruginosa Biofilms by 405-Nanometer-Light-Emitting Diode Illumination. Appl. Environ. Microbiol..

[97] Dos Anjos C., Leanse L.G., Liu X., Miranda H.V., Anderson R.R., Dai T. (2022). Antimicrobial Blue Light for Prevention and Treatment of Highly Invasive Vibrio vulnificus Burn Infection in Mice. Front. Microbiol..

[98] Sousa M., Oliveira I.M., Correia L., Gomes I.B., Sousa C.A., Braga D.F.O., Simões M. (2024). Far-UV-C irradiation promotes synergistic bactericidal action against adhered cells of *Escherichia coli* and *Staphylococcus epidermidis*. Sci. Total Environ..

[99] Nishikawa J., Fujii T., Fukuda S., Yoneda S., Tamura Y., Shimizu Y., Yanai A., Kobayashi Y., Harada K., Kawasaki K. (2024). Far-ultraviolet irradiation at 222 nm destroys and sterilizes the biofilms formed by periodontitis pathogens. J. Microbiol. Immunol. Infect..

[100] Argyraki A., Markvart M., Stavnsbjerg C., Kragh K.N., Ou Y., Bjørndal L., Bjarnsholt T., Petersen P.M. (2018). UV light assisted antibiotics for eradication of in vitro biofilms. Sci. Rep..

[101] Bernbom N., Vogel B.F., Gram L. (2011). Listeria monocytogenes survival of UV-C radiation is enhanced by presence of sodium chloride, organic food material and by bacterial biofilm formation. Int. J. Food Microbiol..

[102] Kim M., Park S.Y., Ha S.-D. (2016). Synergistic effect of a combination of ultraviolet–C irradiation and sodium hypochlorite to reduce *Listeria monocytogenes* biofilms on stainless steel and eggshell surfaces. Food Control.

[103] Srey S., Park S.Y., Jahid I.K., Ha S.-D. (2014). Reduction effect of the selected chemical and physical treatments to reduce *L. monocytogenes* biofilms formed on lettuce and cabbage. Food Res. Int..

[104] Tajik H., Naghili H., Ghasemmahdi H., Moradi M., Badali A. (2015). Effects of *Zataria multiflora* boiss essential oil, ultraviolet radiation and their combination on *Listeria monocytogenes* biofilm in a simulated industrial model. Int. J. Food Sci. Technol..

[105] Roy P.K., Mizan M.F.R., Hossain M.I., Han N., Nahar S., Ashrafudoulla M., Toushik S.H., Shim W.-B., Kim Y.-M., Ha S.-D. (2021). Elimination of *Vibrio parahaemolyticus* biofilms on crab and shrimp surfaces using ultraviolet C irradiation coupled with sodium hypochlorite and slightly acidic electrolyzed water. Food Control.

[106] Bae Y.-M., Lee S.-Y. (2012). Inhibitory effects of UV treatment and a combination of UV and dry heat against pathogens on stainless steel and polypropylene surfaces. J. Food Sci..

[107] Tingpej P., Tiengtip R., Kondo S. (2015). Decontamination Efficacy of Ultraviolet Radiation against Biofilms of Common Nosocomial Bacteria. J. Med. Assoc. Thai..

[108] Murray K.E., Manitou-Alvarez E.I., Inniss E.C., Healy F.G., Bodour A.A. (2015). Assessment of oxidative and UV-C treatments for inactivating bacterial biofilms from groundwater wells. Front. Environ. Sci. Eng..

[109] Bak J., Ladefoged S.D., Tvede M., Begovic T., Gregersen A. (2009). Dose requirements for UVC disinfection of catheter biofilms. Biofouling.

[110] Binns R., Li W., Wu C.D., Campbell S., Knoernschild K., Yang B. (2020). Effect of Ultraviolet Radiation on *Candida albicans* Biofilm on Poly(methylmethacrylate) Resin. J. Prosthodont..

[111] El-Azizi M., Khardori N. (2016). Efficacy of ultraviolet C light at sublethal dose in combination with antistaphylococcal antibiotics to disinfect catheter biofilms of methicillin-susceptible and methicillin-resistant *Staphylococcus aureus* and *Staphylococcus epidermidis* in vitro. Infect. Drug Resist..

[112] Silva-Espinoza B.A., Palomares-Navarro J.J., Tapia-Rodriguez M.R., Cruz-Valenzuela M.R., González-Aguilar G.A., Silva-Campa E., Pedroza-Montero M., Almeida-Lopes M., Miranda R., Ayala-Zavala J.F. (2020). Combination of ultraviolet light-C and clove essential oil to inactivate *Salmonella Typhimurium* biofilms on stainless steel. J. Food Saf..

[113] Jahid I.K., Han N.R., Srey S., Ha S.-D. (2014). Competitive interactions inside mixed-culture biofilms of *Salmonella Typhimurium* and cultivable indigenous microorganisms on lettuce enhance microbial resistance of their sessile cells to ultraviolet C (UV-C) irradiation. Food Res. Int..

[114] Epelle E.I., Amaeze N., Mackay W.G., Yaseen M. (2024). Efficacy of gaseous ozone and UVC radiation against *Candida auris* biofilms on polystyrene surfaces. J. Environ. Chem. Eng..

[115] Malateaux G., Salazar-Gamarra R.E., de Souza Silva J., Pecorari V.G.A., Suffredini I.B., Netto F.P., Neves C.R., Rodrigues de Souza I., de Mello Mesquita A.M., Dib L.L. (2024). Ultraviolet C as a method of disinfecting medical silicone used in facial prostheses: An in vitro study—Part 2. J. Prosthet. Dent..

[116] Richard K.N., Palmer A., Swain G., Hunsucker K.Z. (2025). Assessing the impact of UV-C exposure on pre-existing cultured marine diatom biofilms. Biofilm.

[117] Pousty D., Ma B., Mathews C., Halanur M., Mamane H., Linden K.G. (2024). Biofilm inactivation using LED systems emitting germicidal UV and antimicrobial blue light. Water Res..

[118] Rahman A.H., Mayer B.K., Marshall C.W., Hristova K.R. (2025). UV-LED inactivation of *S. aureus* and *A. baumannii* dual-species biofilm: Insights into the role of interspecies interactions. J. Environ. Chem. Eng..

[119] Bak J., Ladefoged S.D., Tvede M., Begovic T., Gregersen A. (2010). Disinfection of *Pseudomonas aeruginosa* biofilm contaminated tube lumens with ultraviolet C light emitting diodes. Biofouling.

[120] Gora S.L., Rauch K.D., Ontiveros C.C., Stoddart A.K., Gagnon G.A. (2019). Inactivation of biofilm-bound *Pseudomonas aeruginosa* bacteria using UVC light emitting diodes (UVC LEDs). Water Res..

[121] Marasini S., Dean S.J., Swift S., Hussan J.R., Craig J.P. (2025). In vitro anti-biofilm efficacy of therapeutic low dose 265 nm UVC. J. Photochem. Photobiol. B.

[122] Argyraki A., Markvart M., Bjørndal L., Bjarnsholt T., Petersen P.M. (2017). Inactivation of *Pseudomonas aeruginosa* biofilm after ultraviolet light-emitting diode treatment: A comparative study between ultraviolet C and ultraviolet B. J. Biomed. Opt..

[123] Prasad A., Roopesh M.S. (2023). Bacterial biofilm reduction by 275 and 455 nm light pulses emitted from light emitting diodes. J. Food Saf..

[124] Labadie M., Marchal F., Merbahi N., Girbal-Neuhauser E., Fontagné-Faucher C., Marcato-Romain C.-E. (2024). Cell density and extracellular matrix composition mitigate bacterial biofilm sensitivity to UV-C LED irradiation. Appl. Microbiol. Biotechnol..

[125] Pezzoni M., Pizarro R.A., Costa C.S. (2018). Exposure to low doses of UVA increases biofilm formation in *Pseudomonas aeruginosa*. Biofouling.

[126] Li J., Hirota K., Yumoto H., Matsuo T., Miyake Y., Ichikawa T. (2010). Enhanced germicidal effects of pulsed UV-LED irradiation on biofilms. J. Appl. Microbiol..

[127] Angarano V., Smet C., Akkermans S., Watt C., Chieffi A., van Impe J.F.M. (2020). Visible Light as an Antimicrobial Strategy for Inactivation of *Pseudomonas fluorescens* and *Staphylococcus epidermidis* Biofilms. Antibiotics.

[128] Maknuna L., van Tran N., Lee B.-I., Kang H.W. (2023). Inhibitory effect of 405 nm laser light on bacterial biofilm in urethral stent. Sci. Rep..

[129] Buchovec I., Vyčaitė E., Badokas K., Sužiedelienė E., Bagdonas S. (2022). Application of Antimicrobial Photodynamic Therapy for Inactivation of Acinetobacter baumannii Biofilms. Int. J. Mol. Sci..

[130] Astuti S.D., Hafidiana, Rulaningtyas R., Abdurachman, Putra A.P., Samian, Arifianto D. (2020). The efficacy of photodynamic inactivation with laser diode on *Staphylococcus aureus* biofilm with various ages of biofilm. Infect. Dis. Rep..

[131] Halstead F.D., Hadis M.A., Marley N., Brock K., Milward M.R., Cooper P.R., Oppenheim B., Palin W.M. (2019). Violet-Blue Light Arrays at 405 Nanometers Exert Enhanced Antimicrobial Activity for Photodisinfection of Monomicrobial Nosocomial Biofilms. Appl. Environ. Microbiol..

[132] Schafer M.E., McNeely T. (2021). Combining Visible Light and Non-Focused Ultrasound Significantly Reduces Propionibacterium acnes Biofilm While Having Limited Effect on Host Cells. Microorganisms.

[133] Leanse L.G., Zeng X., Dai T. (2021). Potentiated antimicrobial blue light killing of methicillin resistant *Staphylococcus aureus* by pyocyanin. J. Photochem. Photobiol. B.

[134] Giannelli M., Landini G., Materassi F., Chellini F., Antonelli A., Tani A., Nosi D., Zecchi-Orlandini S., Rossolini G.M., Bani D. (2017). Effects of photodynamic laser and violet-blue led irradiation on *Staphylococcus aureus* biofilm and *Escherichia coli* lipopolysaccharide attached to moderately rough titanium surface: In vitro study. Lasers Med. Sci..

[135] Ong J., Godfrey R., Nazarian A., Tam J., Drake L., Isaacson B., Pasquina P., Williams D. (2023). Antimicrobial blue light as a biofilm management therapy at the skin-implant interface in an ex vivo percutaneous osseointegrated implant model. J. Orthop. Res..

[136] Leanse L.G., Goh X.S., Dai T. (2020). Quinine Improves the Fungicidal Effects of Antimicrobial Blue Light: Implications for the Treatment of Cutaneous Candidiasis. Lasers Surg. Med..

[137] Ferrer-Espada R., Liu X., Goh X.S., Dai T. (2019). Antimicrobial Blue Light Inactivation of Polymicrobial Biofilms. Front. Microbiol..

[138] McKenzie K., Maclean M., Timoshkin I.V., Endarko E., MacGregor S.J., Anderson J.G. (2013). Photoinactivation of bacteria attached to glass and acrylic surfaces by 405 nm light: Potential application for biofilm decontamination. Photochem. Photobiol..

[139] Halstead F.D., Ahmed Z., Bishop J.R.B., Oppenheim B.A. (2019). The potential of visible blue light (405 nm) as a novel decontamination strategy for carbapenemase-producing enterobacteriaceae (CPE). Antimicrob. Resist. Infect. Control.

[140] Tsutsumi-Arai C., Arai Y., Terada-Ito C., Takebe Y., Ide S., Umeki H., Tatehara S., Tokuyama-Toda R., Wakabayashi N., Satomura K. (2019). Effectiveness of 405-nm blue LED light for degradation of Candida biofilms formed on PMMA denture base resin. Lasers Med. Sci..

[141] Tsutsumi-Arai C., Arai Y., Terada-Ito C., Imamura T., Tatehara S., Ide S., Wakabayashi N., Satomura K. (2022). Microbicidal effect of 405-nm blue LED light on *Candida albicans* and *Streptococcus mutans* dual-species biofilms on denture base resin. Lasers Med. Sci..

[142] Tsutsumi-Arai C., Arai Y., Terada-Ito C., Imamura T., Tatehara S., Ide S., Shirakawa J., Wakabayashi N., Satomura K. (2022). Inhibitory effect of 405-nm blue LED light on the growth of *Candida albicans* and *Streptococcus mutans* dual-species biofilms on denture base resin. Lasers Med. Sci..

[143] Grangeteau C., Lebleux M., David V., Rousseaux S., Alexandre H., Beney L., Dupont S. (2024). Ultra-high irradiance (UHI) blue light treatment: A promising method for inactivation of the wine spoilage yeast *Brettanomyces bruxellensis*. LWT-Food Sci. Technol..

[144] Olszewska M.A., Dev Kumar G., Hur M., Diez-Gonzalez F. (2023). Inactivation of dried cells and biofilms of *Listeria monocytogenes* by exposure to blue light at different wavelengths and the influence of surface materials. Appl. Environ. Microbiol..

[145] Wang Y., Wu X., Chen J., Amin R., Lu M., Bhayana B., Zhao J., Murray C.K., Hamblin M.R., Hooper D.C. (2016). Antimicrobial Blue Light Inactivation of Gram-Negative Pathogens in Biofilms: In Vitro and In Vivo Studies. J. Infect. Dis..

[146] Treghini C., Insero G., Dell’Accio A., Micieli M., Riccobono E., Valzano F., Fusi F., Rossolini G.M., Pallecchi L., Romano G. (2024). In vitro photoinactivation of *Pseudomonas aeruginosa* and *Staphylococcus aureus* biofilm by a novel multi-dose LED-based illumination method. Photodiagnosis Photodyn. Ther..

[147] de Sousa D.L., Lima R.A., Zanin I.C., Klein M.I., Janal M.N., Duarte S. (2015). Effect of Twice-Daily Blue Light Treatment on Matrix-Rich Biofilm Development. PLoS ONE.

[148] Alves F., Nakada P.J.T., Marques M.J.d.A.M., Rea L.d.C., Cortez A.A., Pellegrini V.d.O.A., Polikarpov I., Kurachi C. (2024). Complete photodynamic inactivation of *Pseudomonas aeruginosa* biofilm with use of potassium iodide and its comparison with enzymatic pretreatment. J. Photochem. Photobiol. B.

[149] Fontana C.R., Song X., Polymeri A., Goodson J.M., Wang X., Soukos N.S. (2015). The effect of blue light on periodontal biofilm growth in vitro. Lasers Med. Sci..

[150] Rosa L.P., da Silva F.C., Viana M.S., Meira G.A. (2016). In vitro effectiveness of 455-nm blue LED to reduce the load of *Staphylococcus aureus* and *Candida albicans* biofilms in compact bone tissue. Lasers Med. Sci..

[151] Wang C., Yang Z., Peng Y., Guo Y., Yao M., Dong J. (2018). Application of 460 nm visible light for the elimination of *Candida albicans* in vitro and in vivo. Mol. Med. Rep..

[152] Moradi M., Fazlyab M., Pourhajibagher M., Chiniforush N. (2022). Antimicrobial action of photodynamic therapy on *Enterococcus faecalis* biofilm using curing light, curcumin and riboflavin. Aust. Endod. J..

[153] Cohen-Berneron J., Steinberg D., Featherstone J.D.B., Feuerstein O. (2016). Sustained effects of blue light on *Streptococcus mutans* in regrown biofilm. Lasers Med. Sci..

[154] Steinberg D., Moreinos D., Featherstone J., Shemesh M., Feuerstein O. (2008). Genetic and physiological effects of noncoherent visible light combined with hydrogen peroxide on Streptococcus mutans in biofilm. Antimicrob. Agents Chemother..

[155] Shany-Kdoshim S., Polak D., Houri-Haddad Y., Feuerstein O. (2019). Killing mechanism of bacteria within multi-species biofilm by blue light. J. Oral Microbiol..

[156] Chebath-Taub D., Steinberg D., Featherstone J.D.B., Feuerstein O. (2012). Influence of blue light on *Streptococcus mutans* re-organization in biofilm. J. Photochem. Photobiol. B.

[157] Vaknin M., Steinberg D., Featherstone J.D., Feuerstein O. (2020). Exposure of *Streptococcus mutans* and *Streptococcus sanguinis* to blue light in an oral biofilm model. Lasers Med. Sci..

[158] Liang J., Huang T.Y., Li X., Gao Y. (2023). Germicidal effect of intense pulsed light on *Pseudomonas aeruginosa* in food processing. Front. Microbiol..

[159] Garvey M., Rabbitt D., Stocca A., Rowan N. (2015). Pulsed ultraviolet light inactivation of *Pseudomonas aeruginosa* and *Staphylococcus aureus* biofilms. Water Environ. J..

[160] Montgomery N.L., Banerjee P. (2015). Inactivation of Escherichia coli O157:H7 and *Listeria monocytogenes* in biofilms by pulsed ultraviolet light. BMC Res. Notes.

[161] Garvey M., Andrade Fernandes J.P., Rowan N. (2015). Pulsed light for the inactivation of fungal biofilms of clinically important pathogenic *Candida* species. Yeast.

[162] Chick H. (1908). An Investigation of the Laws of Disinfection. J. Hyg..

[163] Watson H.E. (1908). A Note on the Variation of the Rate of Disinfection with Change in the Concentration of the Disinfectant. J. Hyg..

